# Self-reported changes in sleep patterns and behavior in children and adolescents during COVID-19

**DOI:** 10.1038/s41598-022-24509-7

**Published:** 2022-11-27

**Authors:** Kathrin Bothe, Manuel Schabus, Esther-Sevil Eigl, Reinhold Kerbl, Kerstin Hoedlmoser

**Affiliations:** 1 Laboratory for Sleep, Cognition and Consciousness Research, Department of Psychology, Centre for Cognitive Neuroscience, University of Salzburg, Salzburg, Salzburg, Austria; 2 Department of Pediatrics and Adolescent Medicine, Leoben Regional Hospital, Leoben, Styria Austria

**Keywords:** Circadian rhythms and sleep, Paediatric research, Psychology, Human behaviour

## Abstract

The COVID-19 pandemic and lockdowns worldwide forced children and adolescents to change and adapt their lives to an unprecedented situation. Using an online survey, we investigated whether they showed changes in sleep quality and other related factors due to this event. Between February 21st, 2021 and April 19th, 2021, a total of 2,290 Austrian children and adolescents (6–18 years) reported their sleep habits and quality of sleep as well as physical activity, daylight exposure and usage of media devices during and, retrospectively, before the pandemic. Results showed an overall delay of sleep and wake times. Almost twice as many respondents reported having sleeping problems during the pandemic as compared to before, with insomnia, nightmares and daytime sleepiness being the most prevalent problems. Furthermore, sleeping problems and poor quality of sleep correlated positively with COVID-19 related anxiety. Lastly, results showed a change from regular to irregular bedtimes during COVID-19, higher napping rates, a strong to very strong decrease in physical activity and daylight exposure, as well as a high to very high increase in media consumption. We conclude that the increase in sleeping problems in children and adolescent during COVID-19 is concerning. Thus, health promoting measures and programs should be implemented and enforced.

## Introduction

Sleep is an important factor for our general well-being. Healthy sleep aids the regulation of behavior, emotions, and stress^1,2^. Sleep problems on the other hand can negatively affect these domains and are also likely to have an impact on cognition, physical health, and academic performance^3^. Due to rapid developmental changes (e.g., cortical reorganization processes in the maturing brain), children and adolescents are at heightened risk for the onset of sleep and mental health issues^4,5^. In fact, it has been shown that sleeping problems in children and adolescents are linked to behavioral (e.g., self-regulation, conduct, attention) and emotional (e.g., depression, anxiety, stress) problems^6–8^. Furthermore, longitudinal studies indicate that sleeping problems during childhood and adolescence heighten the risk for the development of psychopathology—particularly anxiety and depression disorders^9–12^, but also attention problems and aggression^7^—later in life. Thus, promoting and maintaining an optimal quality of sleep during this vulnerable phase of life seems to be of special importance.

The COVID-19 pandemic and lockdowns worldwide forced people to change and adapt their lives to an unprecedented situation. Since most activities that typically occupy youths’ lives have been highly restricted (e.g., in-person teaching, community-based and leisure activities, socializing with peers), children and adolescents may be especially affected by anti-COVID-19 measures^13,14^. Thus, the pandemic and the implemented restrictions can have serious consequences on emotional and physical well-being as well as on sleep^15,16^. For example, having to cope with major changes in routines (e.g., shift in the regularity of daily schedules; homeschooling) and worrying about one’s own or the health of others likely causes a lot of stress and anxiety^14,17^. Via increased arousal levels or vigilance, this may lead to difficulties falling asleep or to an increase in the number of nocturnal awakenings, thereby shortening sleep and lowering its quality^18,19^. Additionally, the circadian sleep drive may be lower after spending less time under natural sunlight and fresh air, thus possibly delaying sleep onset^20^. A reduction in physical activity and weight gains due to long phases of home confinement may also have a negative impact on sleep quality and well-being^21,22^. Furthermore, the use of screen-based media devices emitting high amounts of blue light close to bedtime may suppress melatonin secretion and the contents (e.g., social media, news, movies) consumed via those devices can make falling asleep more difficult^23^.

Thus, the potential for sleeping problems to emerge or worsen during this challenging period is high. The majority of studies and surveys regarding sleep in children and adolescents (especially in the age range between 6 and 14 years) during COVID-19 seem to be parent-reported^24^. However, research suggests that the validity of reports is higher when individuals report their own perceptions. This has also been shown for sleep-related measures (e.g., sleep quality and duration, sleep timing) in children and adolescents^25–27^. Self-reported data can facilitate the understanding of internal experiences, e.g., regarding well-being, health and emotions and have been shown to be sufficiently reliable in children from about 6 years of age and onwards^28,29^.

Hence, we designed a self-reported online survey that aimed at assessing whether and to which extent children and adolescents (6–18 years) subjectively perceived changes in their sleep patterns and behavior during COIVD-19 compared to before. The survey included questions regarding changes in bed- and wake times (e.g., due to changes in daily structure), changes in sleep quality as well as in the frequency, duration, and burden of sleeping problems (e.g., due to anxiety related to COVID-19). Furthermore, additional factors (e.g., restriction of outdoor activities) that could have affected their sleep during COVID-19 were assessed.

## Results

The complete results (descriptive and statistics) can be found in the Supplementary Information.

### Demographic data

Out of 5483 participants^17,30^, 2565 participants continued with the sleep part of the survey. Since 275 participants aborted before completing the survey, the final sample consisted of 2290 participants: 1439 females (62.8%), 818 males (35.7%) and 33 diverse (1.4%) with the following age distribution: 6–10 years 375 (16.4%), 11–14 years 962 (42.0%), and 15–18 years 953 (41.6%). Descriptive statistics of the sample are reported in Table 1. Please note that due to the small sample size of participants identifying as diverse, only participants identifying as female and male were considered for further analyses.

**Table 1:** Demographic characteristics of participants.

Response option	6–10 years					11–14 years					15–18 years				
	n	N	%	95% CI		n	N	%	95% CI		n	N	%	95% CI	
				Lower	Upper				Lower	Upper				Lower	Upper
**Sex**															
Male	188	375	50.1	44.96	55.31	376	952	39.5	36.38	42.68	254	930	27.3	24.47	30.30
Female	187	375	49.9	44.69	55.04	576	952	60.5	57.32	63.63	676	930	72.7	69.70	75.53
**In which state do you live?**															
Burgenland	5	375	1.3	0.43	3.08	21	952	2.2	1.37	3.35	20	930	2.2	1.32	3.30
Carinthia	18	375	4.8	2.87	7.48	33	952	3.5	2.40	4.83	13	930	1.4	0.75	2.38
Lower Austria	39	375	10.4	7.50	13.94	85	952	8.9	7.19	10.92	140	930	15.1	12.82	17.52
Upper Austria	40	375	10.7	7.73	14.24	90	952	9.5	7.67	11.49	96	930	10.3	8.44	12.46
Salzburg	59	375	15.7	12.20	19.82	145	952	15.2	13.01	17.67	173	930	18.6	16.15	21.26
Styria	104	375	27.7	23.26	32.56	331	952	34.8	31.74	37.89	259	930	27.8	24.99	30.85
Tyrol	14	375	3.7	2.06	6.18	33	952	3.5	2.40	4.83	38	930	4.1	2.91	5.57
Vorarlberg	13	375	3.5	1.86	5.86	22	952	2.3	1.45	3.48	28	930	3.0	2.01	4.32
Vienna	83	375	22.1	18.03	26.68	192	952	20.2	17.66	22.86	163	930	17.5	15.14	20.13
**What school are you attending?**															
Elementary school: Volksschule	373	375	99.5	98.09	99.94										
School for people with special needs	2	375	0.5	0.06	1.91	5	952	0.5	0.17	1.22	3	930	38.0	34.83	41.16
Midldle school: AHS Unterstufe						250	952	26.3	23.49	29.18	8	930	0.9	0.37	1.69
Middle school: Gymnasium						243	952	25.5	22.78	28.42	63	930	41.0	37.79	44.21
Middle school: Neue Mittelschule						382	952	40.1	37.00	43.32	30	930	2.4	1.49	3.56
High school: BHS (HTL, HAK, HLW, BAFEP)						26	952	2.7	1.79	3.98	381	930	5.3	3.92	6.91
High school: BMS (Fachschule, Handelsschule)						4	952	0.4	0.12	1.07	22	930	6.8	5.24	8.58
High school: Berufsschule und Lehre						2	952	0.2	0.03	0.76	49	930	3.2	2.19	4.57
High school: AHS Oberstufe						34	952	3.6	2.49	4.96	353	930	2.3	1.40	3.43
High school: Polytechnische Schule						6	952	0.6	0.23	1.37	21	930	0.3	0.07	0.94

*CI, 95% *confidence interval with lower and upper border; *empty spaces* response option was not chosen in this age group.

### Bed- and wake times

**Figure 1:**
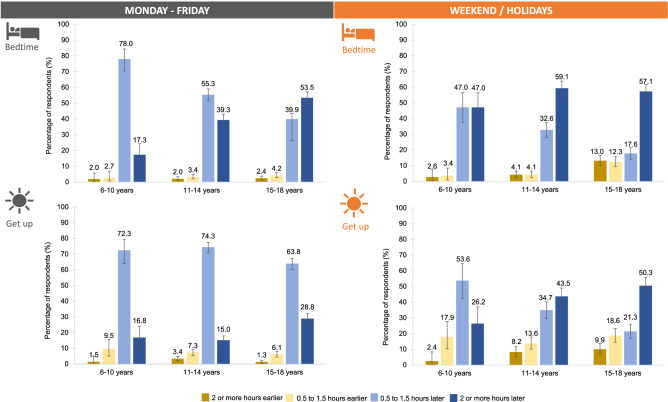
Changes in sleep rhythm. Delay in bed- and wake times in children and adolescents during COVID-19 compared to before. During the week as well as on weekends (more pronounced), children and adolescents reported later bedtimes. However, wake times, especially at weekdays, were not delayed to the same extent as bedtimes. Additionally, the delay in bed- and wake times increased with age, i.e., 15–18-year-olds reported the strongest delays.

On average, 63% reported a change in bed- and wake times during the week. Regarding the weekend, only 41% reported a change in bedtimes and 31% in wake times. Overall, 6–10-year-olds always reported significantly smaller changes than 11–14- and/or 15–18-year-olds (Table S2). With reference to the direction of change, there was a strong delay in bed- and wake times in all age groups (Fig. 1, Table S1). Especially during the week, 15–18-year-olds reported significantly later bedtimes and wake times than 6–10-year and 11–14-year-olds (Table S2).

Among the 6–10-year-olds, the delay mainly ranged within 0.5 to 1.5 h and increased further with age– for example, 57% of the 15–18-year-old reported going to bed 2 or more hours later on weekends. However, wake times, particularly during the week, were not delayed to the same extent as bedtimes (e.g., 11–14 years: bedtime 2 or more hours later: 39.7%; wake time 2 or more hours later: 15.0%; difference of 24.7 percentage points).

11–14-year-old females went to bed significantly later during the week and on weekends while 15–18 year-old males went to bed and tended to get up later on weekends than their female counterparts (Table S3).

### General sleep quality, sleeping problems and COVID-19 related anxiety

**Figure 2:**
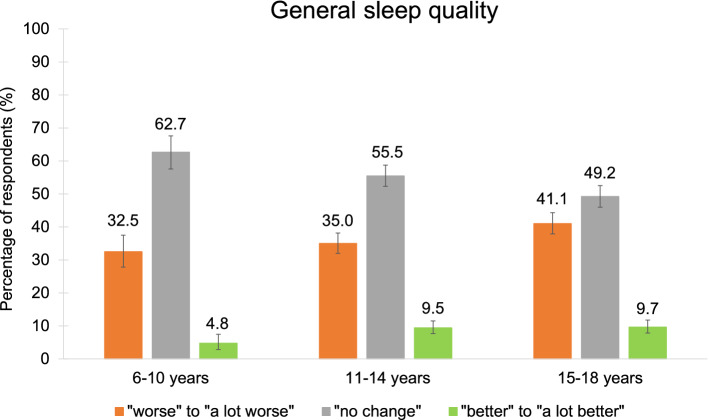
Change in general sleep quality. Around one third of children and adolescents reported to sleep “worse” or “a lot worse” while more than half of the participants experienced “no change” in their quality of sleep compared to before the pandemic.

On average, 56% of the participants noticed a change in general sleep quality with most of them sleeping “worse” or “a lot worse” than before the pandemic (Fig. 2, Table S4). Overall, females rated their sleep quality worse than males (Table S6).

**Figure 3:**
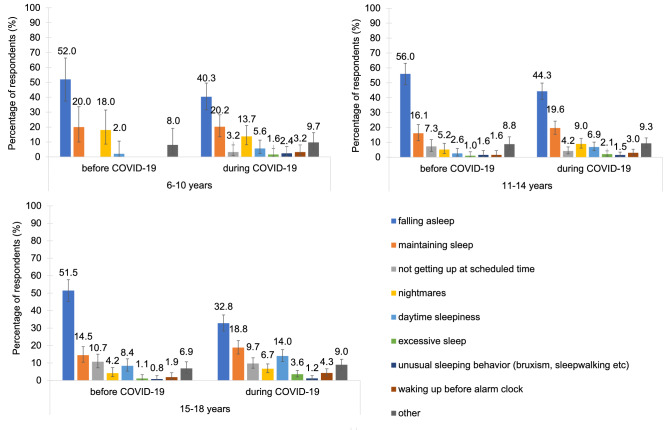
Types of sleeping problems. The distribution of types of sleeping problems in children and adolescents was similar before and during the pandemic.

Before COVID-19, the youngest participants (6–10 years) reported the least sleeping problems (13.3%), followed by the 11–14-year-olds (20.3%) and the 15–18-year-olds (28.2%). Across all age groups, difficulties falling asleep and maintaining sleep were the most common (Fig. 3). In the 6–10-year-olds, nightmares were also relatively common. In the 11–14-year-olds and 15–18-year-olds, difficulties getting up at the scheduled time and daytime sleepiness were the third and fourth common sleeping difficulties. Regarding the duration of sleeping problems, all age groups showed a similar pattern of roughly one third having the problems for the duration of 0–6 months, between 6 months and 2 years, or between 2 and more than 5 years (Table S4). In terms of burden, 6–10-year-olds mainly reported their sleeping difficulties as being a little burdensome or moderately to severely burdensome while the majority of the 11–18-year-olds perceived them as moderately to severely burdensome (Table S4).

**Figure 4:**
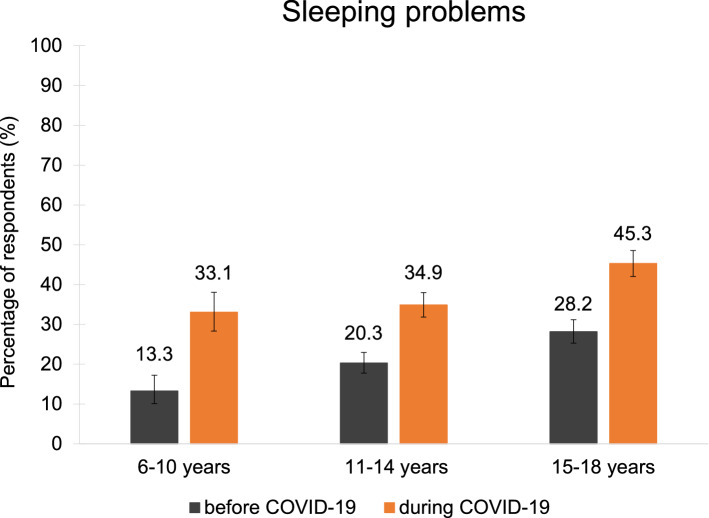
Change in sleeping problems. Children and adolescents reported almost twice as many (M = 1.9 times as much) sleeping problems during COVID-19 compared to before.

Compared to before COVID-19, all age groups reported a considerable and significant increase in sleeping problems during the pandemic (Fig. 4, Tables S4, S8): 6–10 years: from 13.3% to 33.1% (+ 19.8 percentage points); 11–14 years: 20.3% to 34.9% (+ 14.6 percentage points); 15–18 years: 28.2 to 45.3% (+ 17.1 percentage points).

Similar to before the pandemic (Fig. 3, Table S4), the most common sleeping problems during COVID-19 were difficulties falling asleep and maintaining sleep. Furthermore, nightmares were the third most common issue in the 6–10-year-olds as well as in the 11–14-year-olds. In the 15–18-year-olds, difficulties falling asleep were still the most common problem. However, difficulties maintaining sleep, daytime sleepiness and difficulties getting up at the desired time became a lot more prominent during COVID-19. In all age groups, sleeping problems during COVID-19 mostly persisted between 1 and 6 months or more than 6 months. In terms of burden, 6–10-year-olds showed an increase from 42.0% to 54.8% in the moderate to severe category (Tables S4).

**Figure 5:**
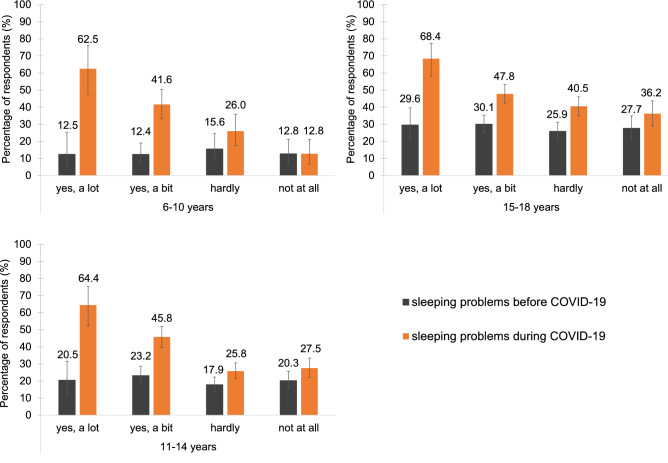
Sleeping problems and COVID-19 related anxiety. Reports of sleeping problems during COVID-19 were more frequent in children and adolescents being more anxious (“yes, a lot”) of COVID-19 than those being less anxious (“not at all”).

Furthermore, correlations (Table 2) and nonparametric statistics (Tables S7, S8) showed that a stronger COVID-19 related anxiety was significantly associated with a worse quality of sleep and a higher amount of sleeping problems. Across all age groups, 64% of the very anxious participants (“yes, a lot”) reported to have a reduced (“worse”; “a lot worse”) sleep quality during COVID-19. This percentage gradually decreased throughout the response categories. A very similar pattern emerged for sleeping problems, i.e., 65.1% of the very anxious participants reported having sleeping problems. Again, this percentage decreased throughout the remaining response categories (Fig. 5, Tables S7, S8).

**Table 2:** Correlation coefficients (Kendall’s Tau, *τ*_*b*_) for COVID-19 related anxiety and general sleep quality as well as sleeping problems during COVID-19.

COVID-19 related anxiety	Age group															Overall				
	6–10 years					11–14 years					15–18 years					6–18 years				
	n	*τ*_*b*_	p	95% CI		n	*τ*_*b*_	p	95% CI		n	*τ*_*b*_	p	95% CI		n	*τ*_*b*_	p	95% CI	
				Lower	Upper				Lower	Upper				Lower	Upper				Lower	Upper
General sleep quality	345	0.34	** < 0.001**	0.28	0.42	952	0.19	** < 0.001**	0.15	0.23	930	0.14	** < 0.001**	0.10	0.18	2257	0.20	** < 0.001**	0.18	0.23
Female	187	0.35	** < 0.001**	0.27	0.47	576	0.21	** < 0.001**	0.16	0.27	676	0.16	** < 0.001**	0.11	0.21	1439	0.20	** < 0.001**	0.17	0.24
Male	188	0.33	** < 0.001**	0.25	0.44	376	0.16	** < 0.001**	0.09	0.22	254	0.09	0.086	0.01	0.18	818	0.16	** < 0.001**	0.12	0.21
Sleeping problems	345	0.31	** < 0.001**	0.25	0.39	953	0.19	** < 0.001**	0.15	0.23	930	0.15	** < 0.001**	0.10	0.18	2257	0.20	** < 0.001**	0.18	0.23
Female	187	0.29	** < 0.001**	0.20	0.40	576	0.18	** < 0.001**	0.13	0.24	676	0.16	** < 0.001**	0.11	0.21	1439	0.19	** < 0.001**	0.16	0.23
Male	188	0.33	** < 0.001**	0.24	0.44	376	0.18	** < 0.001**	0.12	0.25	254	0.08	**0.017**	0.00	0.16	818	0.19	** < 0.001**	0.15	0.24

τb 0.07–0.19 small effect; τb 0.20–0.33 medium effect; τb 0.34–0.49 strong effect; 95%CI: 95% confidence interval with lower and upper border.

Significant values are in bold.

Comparisons of sleeping problems, especially in the 6–10- and 11–14-year-olds, further revealed that participants with the highest COVID-19 related anxiety also showed the strongest increases (i.e., largest effect sizes) in sleeping problems from before to during COVID-19 (Table S8). In the 15–18-year-olds, a general increase in sleeping problems, regardless of COVID-19 related anxiety was observed. However, similar to the other age groups, effect sizes were strongest for the most anxious participants.

Overall, sleeping problems and worsening of general sleep quality were significantly higher in female as compared to male participants (Table S8). However, effect sizes indicated rather small effects. Regarding COVID-19 related anxiety and sleeping problems, results show no significant differences between males and females in the “a lot” and “a bit” anxious category, thus indicating a similar level of sleeping problems in both sexes related to COVID-19 anxiety.

### Regular bedtimes, time in bed and daytime sleepiness/napping

**Figure 6:**
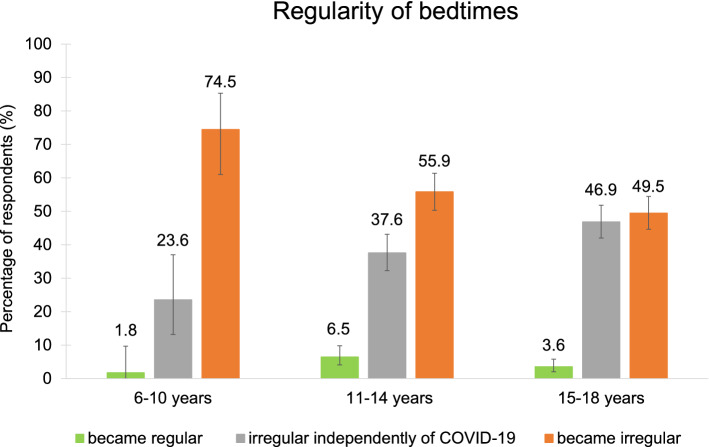
Changes in regularity of bedtimes. Especially for 6–10-year-olds, bedtimes became irregular during COVID-19.

Comparing the regularity of their bedtime during COVID-19 to before, most participants stated that it was regular before and became irregular during the pandemic (Fig. 6, Table 3). Age groups differed significantly in the regularity of bedtimes, i.e., the 6–10-year-olds reported more regular bedtimes than the 11–14- and 15–18-year-olds (Table S9). Interestingly, around 50% of the 15–18-year-olds reported having irregular bedtimes independently of COVID-19 while the remaining half experienced irregular bedtimes only with the emergence of the pandemic.

**Table 3:** Changes in time spent in bed, regularity of bedtimes and daytime sleep/napping.

Response option	6–10 years					11–14 years					15–18 years				
	n	N	%	95% CI		n	N	%	95% CI		n	N	%	95% CI	
				Lower	Upper				Lower	Upper				Lower	Upper
**How did the time you usually spent in bed change?**															
(1) A lot shorter	5	375	1.3	0.43	3.08	31	952	3.3	2.22	4.59	24	929	2.6	1.66	3.82
(2) Considerably shorter	7	375	1.9	0.75	3.81	47	952	4.9	3.65	6.51	55	929	5.9	4.49	7.64
(3) Slightly shorter	49	375	13.1	9.83	16.90	139	952	14.6	12.42	17.01	104	929	11.2	9.24	13.40
(4) No change	197	375	52.5	47.34	57.68	253	952	26.6	23.79	29.50	128	929	13.8	11.63	16.16
(5) Slightly longer	92	375	24.5	20.26	29.21	296	952	31.1	28.16	34.14	307	929	33.0	30.03	36.18
(6) Considerably longer	19	375	5.1	3.08	7.80	115	952	12.1	10.08	14.32	195	929	21.0	18.41	23.75
(7) A lot longer	6	375	1.6	0.59	3.45	71	952	7.5	5.87	9.31	116	929	12.5	10.43	14.79
**Do you go/ did you go to bed at the same time every day (+ /- 30 min)? My bedtime…**															
(1) Yes	320	375	85.3	81.34	88.76	630	952	66.2	63.07	69.18	511	930	54.9	51.68	58.18
(2) No	55	375	14.7	11.24	18.66	322	952	33.8	30.82	36.93	419	930	45.1	41.82	48.32
(1) Was regular before and got irregular during COVID-19	41	55	74.5	61.00	85.33	180	322	55.9	50.29	61.40	207	418	49.5	44.63	54.42
(2) Was irregular before and got regular during COVID-19	1	55	1.8	0.05	9.72	21	322	6.5	4.08	9.80	15	418	3.6	2.02	5.85
(3) Is irregular independently of COVID-19	13	55	23.6	13.23	37.02	121	322	37.6	32.27	43.12	196	418	46.9	42.02	51.80
**Do you usually sleep during the day?**															
(1) Yes	6	375	1.6	0.59	3.45	88	952	9.2	7.48	11.26	258	930	27.7	24.89	30.74
(2) No	369	375	98.4	96.55	99.41	864	952	90.8	88.74	92.52	672	930	72.3	69.26	75.12
(1) I nap from time to time	5	6	83.3	35.88	99.58	56	88	63.6	52.69	73.63	154	258	59.7	53.43	65.73
(2) I nap regularly				0.00	0.00	7	88	8.0	3.26	15.71	46	258	17.8	13.36	23.06
(3) I often fall asleep unintentionally during the day	1	6	16.7	0.42	64.12	25	88	28.4	19.30	39.02	58	258	22.5	17.54	28.07
**Did the frequency of napping change during COVID-19?**															
(1) Yes	22	375	5.9	3.71	8.75	154	952	16.2	13.89	18.67	346	930	37.2	34.09	40.40
(2) No	353	375	94.1	91.25	96.29	798	952	83.8	81.33	86.11	584	930	62.8	59.60	65.91
(1) I nap a lot less than before	3	22	13.6	2.91	34.91	6	154	3.9	1.44	8.29	19	346	5.5	3.34	8.44
(2) I nap less than before	3	22	13.6	2.91	34.91	10	154	6.5	3.16	11.62	34	346	9.8	6.90	13.46
(3) I nap more than before	12	22	54.5	32.21	75.61	98	154	63.6	55.51	71.23	202	346	58.4	52.99	63.63
(4) I nap a lot more than before	4	22	18.2	5.19	40.28	40	154	26.0	19.25	33.65	91	346	26.3	21.74	31.28

*CI 95%* confidence interval with lower and upper border, *empty spaces* response option was not chosen in this age group.

Concerning the time spent in bed (Q 9; i.e., not total sleep time), data indicated prolonged times spent in bed especially for the 11–14- as well as the 15–18-year-old during COVID-19 as compared to before (Table 3, Table S9).

The frequency of daytime naps in habitual nappers (M = 20%) changed during COVID-19, i.e., most participants reported to nap “more” to “a lot more” than before the pandemic. Asked about the circumstances of their napping, 28.4% of the 11–14 and 22.5% of the 15–18-year-olds-reported to often fall asleep unintentionally during the day (Table 3). Overall, females in the 11–14 and 15–18 years-old groups napped significantly more than their male counterparts (Table S10).

### Physical activity, daylight exposure, usage of media devices

**Figure 7:**
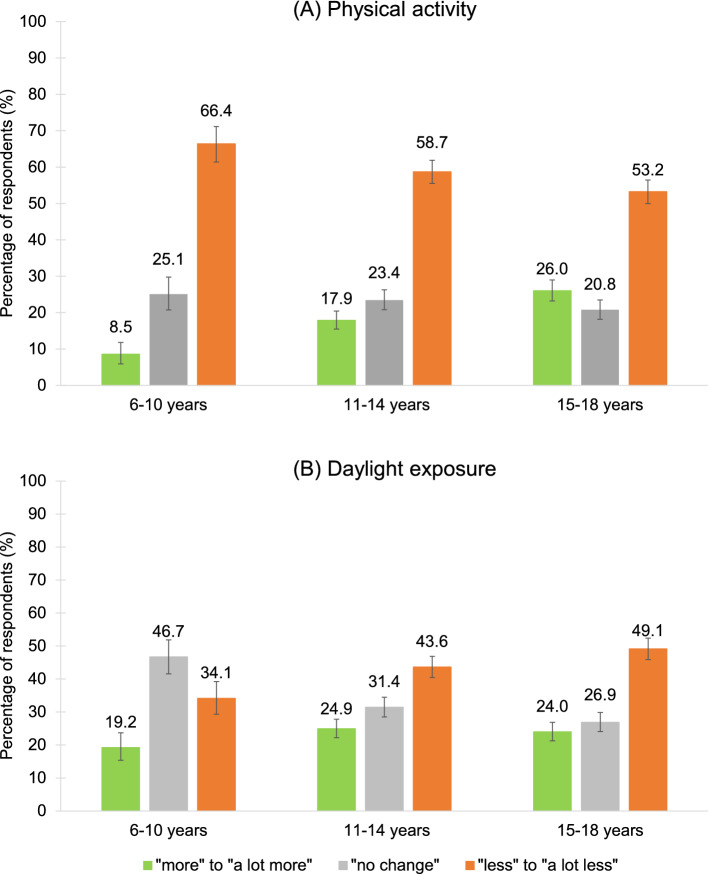
Changes in (**A**) physical activity and (**B**) daylight exposure. (**A**) More than half of the participants were “less” or “a lot less” active during COVID-19 compared to before. With almost 70%, the youngest age group (6–10 years) showed the highest reduction of activity levels. (**B**) An average of 42% experienced “less” or “a lot less” daylight exposure during COVID-19.

Across all age groups, the amount of physical activity changed in more than 75% of participants (Fig. 7A, Table S11), i.e., all age groups reported being “less” to “a lot less active” than before COVID-19 with the 6–10-year-olds being the least active (Table S12). However, an average of 23% also reported being “more” or “a lot more” active.

Regarding daylight exposure, a gradual change across age groups could be observed, i.e., the older the participant the stronger the change in daylight exposure (Tables S11, S12). Most participants, especially 15–18-year-old males (Tables S11, S12), experienced “less” to “a lot less” daylight exposure during COVID-19 (M = 42%). However, an average of 22% of respondents also reported an increase (“more” to “a lot more”) in daylight exposure (Fig. 7B, Table S11).

Usage of media devices during COVID-19 increased considerably and with age. On average, 83% (6–10 years: 74%; 11–14 years: 85%; 15–18 years: 90%) spent more time in front of their smartphones, gaming consoles, PCs, or other media devices (Tables S11–S13).

## Discussion

With this survey, we wanted to evaluate whether Austrian children and adolescents (6–18 years) showed changes in sleep patterns and behavior during COVID-19. Our results are in line with children and adolescent studies from other countries and show an overall delay of sleep and wake times in about 50% of the sample during the week as well as on weekends^24,30–40^. Furthermore, sleeping problems increased significantly during COVID-19. Compared to before, almost twice as many children and adolescents reported having sleeping problems during COVID-19. Moreover, COVID-19 related anxiety seemed to be an important factor for general sleep quality and sleeping problems. Our data show that the higher the COVID-19 related anxiety the worse the perception of general sleep quality and the more sleeping problems were reported. In addition, bedtimes became irregular in most of the participants, time in bed increased as well as the frequency of naps and daytime sleep. Lastly, children and adolescents were less active, spent less time in daylight and showed a considerable increase in the usage of media devices.

Our results regarding a delay in bed- and wake times are in line with previous surveys examining samples with similar age ranges^24,31,32–40^. Our data display a gradual increase towards later bed- and wake times with age, i.e., most 6–10-year-olds reported a delay of 0.5 to 1.5 h while the majority of the 15–18-year-olds show a considerable delay of 2 or more hours. The differences across age groups may be explained by the extent of parental monitoring, i.e., parents might be stricter with the youngest (e.g., enforcing set bedtimes) while adolescents are more independent in choosing their bed- and wake times^41^. Furthermore, humans experience a circadian phase delay around the onset of puberty^42^, i.e., due to developmental changes they shift to a later-typed chronotype that favors late nights and late mornings^43^. Thus, especially for adolescents, home confinement may have been an opportunity to shift their sleep schedules away from societal and towards their own circadian preferences^14^, thereby decreasing social jetlag during the pandemic^44,45^.

Similar to other studies, our data show a marked increase in the usage of media devices within all age groups during the pandemic^35,38,46^. These devices usually come with light emitting diode (LED)- displays that are highly enriched with a blue light component (446–483 nm)^47^. Since shorter wavelength light or blue light is the most effective in suppressing melatonin^48^, the usage of media devices before bedtime can lead to a phase delay by slowing down its secretion^49^, i.e., people feel/stay more alert and less sleepy for extended periods of time. Due to their increased sensitivity to light, this could be especially detrimental for the sleep regulation of pre- to mid- pubertal children (9.1–14.7 years)^50,51^. Additionally, social media use and screen time among children and adolescents have consistently been associated with poorer sleep patterns, i.e., later sleep onset, shorter sleep durations as well as increased sleeping problems like trouble falling asleep after nighttime awakening^23,52^.

**Figure 8:**
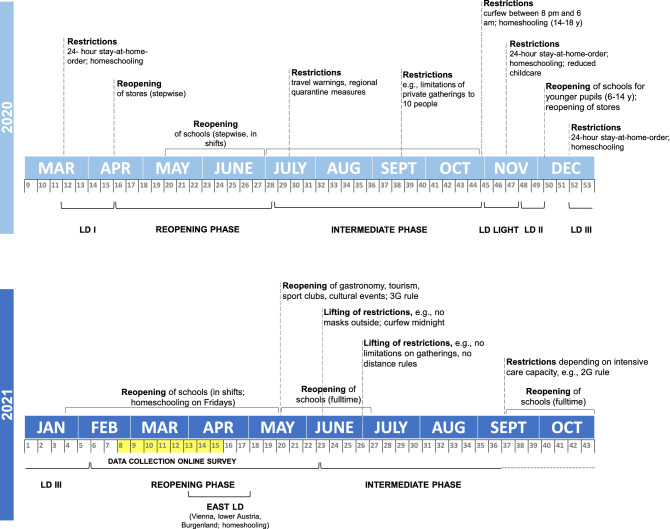
Timeline of COVID-19 related measures in Austria from March 2020 to September 2021 including information about lockdowns (LD) and school closures/reopenings. Highlighted in yellow is the time window of data collection for the online survey.

While both bed- and wake times showed a delay, the shift in wake times did not seem to be equal to the shift in bedtimes, i.e., participants reported an overall smaller delay in wake times during weekdays as well as at the weekend compared to the delay in bedtimes. Paradoxically, this might result in shortened sleep durations (i.e., shorter total sleep time) although the participants would have been more flexible to attune their sleep schedules to their individual needs during COVID-19. A shortening of sleep duration has also been reported in studies with 11–16- and 6–17-year-old Chinese children and adolescents as well as in an Italian sample^37,53,54^. However, several studies also observed an increase in sleep durations during the pandemic^36,37–40^. In addition, wake times of children and adolescents are more often determined by external factors^55^. Since in-person teaching still took place in Austria (at least during the time data from this survey was collected; Fig. 8) and the majority of participants went to school 1–5 times per week (homeschooling usually on Fridays or in the regions affected by the “East Lockdown”), this might be an explanation for the smaller delays in wake times during weekdays^17^. Furthermore, a mixture of hybrid, online and in-person teaching may have led to a greater night-to-night variability in sleep–wake patterns like it has recently been shown in a sample of U.S. students (13–18 years)^56^. However, since we did not assess the actual bedtimes, wake times and sleep durations before and during the pandemic, the assumption that the pandemic situation might have intensified this effect cannot be further verified.

Around one third of the participants reported a drop in general sleep quality and almost twice as many respondents reported having sleeping problems (38.0% vs. 20.9%). Similar to other studies, females rated their sleep quality significantly worse than males and also reported more sleeping problems^36,57^. However, effect sizes for these differences were rather small. Further, insomnia symptoms (difficulties falling asleep and maintaining sleep), nightmares and daytime sleepiness were the most prevalent problems (similar ranking as before the pandemic)^24,37,38,40,58,59^. Compatible with that are the reports regarding an increase in naps and the time spent in bed, thus possibly further facilitating an overall disruption of daily structure resulting in irregular bedtimes.

There might be multiple reasons for the increase in sleeping problems during COVID-19: First, our data show a considerable decrease of physical activity in around three quarters of the participants with the youngest (6–10 years old) showing the strongest reduction (88.6% answered to be “less” to “a lot less” active). This is especially concerning since the Austrian recommendation for physical activity for children and adolescents between 6 and 18 years is 60 min or more per day with moderate to vigorous intensity^60^. Sedentary behavior and obesity do not only have detrimental short- and long-term effects on physical (e.g., cardiovascular disease, high fasting insulin levels/ type-2 diabetes, metabolic syndrome, cancer, musculoskeletal disorders)^60–63^ and mental health (e.g., depression, stress, anxiety, low self-esteem)^64,65^ but also on the quality of sleep. For example, it has been associated with an increased risk for insomnia, shorter sleep durations, disturbed or interrupted sleep as well as increased daytime sleepiness^65–69^. Our data provide indications towards these negative effects and support findings from other studies conducted during the pandemic^35,46^. In addition, regular physical exercise and sleep strengthen the immune system and can thus be an important tool against COVID-19 and other infections^69–73^.

A second reason for the increase in sleeping problems might be the decrease in daylight exposure. Across all age groups, 42.2% experienced “less” to “a lot less” daylight exposure during the pandemic. Daylight is a natural “Zeitgeber” and helps to synchronize the circadian system with the sleep–wake cycle^74,75^. It has been shown that low light exposure during the day sensitizes the circadian system for light exposure during the evening hours, i.e. (artificial) light exposure around and after dusk leads to greater melatonin suppression and a delay of sleep onset^75–78^. Thus, especially the increase in difficulties falling asleep and getting up at the desired time the next morning may have been partly influenced by insufficient daylight exposure.

Further, our data show a positive correlation between sleeping problems and COVID-19 related anxiety with the strongest effects being detected in the youngest participants (6–10 years). This is concerning since the co-occurrence of sleeping problems and anxiety/depression during childhood and adolescence is indicative of long-term psychological difficulties in adulthood^78–81^. For example, a longitudinal study examining sleeping problems in childhood (mean age 10.3 years) and 18 years later showed that subjects with sleeping problems during childhood were 4.51 times more likely to have persistent internal symptoms (depression, anxiety, somatic complaints) in adulthood^82^. Apart from long-term effects, anxiety can also have immediate effects on sleep, e.g., making it more difficult to fall asleep and/or stay asleep due to nighttime fears and nightmares. Persistent rumination, worrying and negative thoughts can lead to heightened levels of physiological arousal, thus potentially delaying/ disturbing sleep and leading to a perpetual cycle of lack of sleep, daytime fatigue, impaired academic and social functioning as well as an impaired ability to regulate mood and emotional responses^83^. Our results also fit well with reports from adult studies showing that reduced sleep quality during COVID-19 was most prevalent in people with elevated symptoms of anxiety and depression^44,83–87^ as well as with low social support^88^. Interestingly, females and males did not show significant differences for COVID-19 related anxiety and sleeping problems thus indicating that both sexes were equally affected.

Lastly, our data should be interpreted in light of some limitations. First, we used a self-designed, thus not validated, questionnaire and our data was not derived from a randomized sample. Hence, there might be potential biases regarding sample composition, e.g., motivation to finish both parts of the survey which may not allow for direct comparisons to a norm or other studies. Nevertheless, we think our approach was highly justified given the unprecedented COVID-19 situation which necessitated specific questions related to the COVID-19 burdens children and adolescent may have suffered from. Third, the online survey could only reach participants being able to use electronic devices. However, due to COVID-19 restrictions (e.g., lockdown, social distancing), online recruitment represented a unique way of accessing a large number of participants within a limited time window. Furthermore, we used retrospective questions to compare the situation during COVID-19 with the time before lockdowns. Although this method seems to be sufficiently reliable^89^, we cannot rule out the occurrence of recall bias, especially in our youngest participants (6 years). Additionally, they may not have been able to understand all of the questions correctly. Thus, although 6-year-olds should be able to answer questions about their sleep^25–29^, these data need to be interpreted with caution. However, excluding the 6-year-olds from the analyses would not have affected the overall results in the respective age group. Data further seemed to be valid since they were in line with the patterns observed in the other age groups (11–14 years, 15–18 years). Finally, we want to highlight that the sample size, including a larger number of very young children, is a strength of the study, together with the interesting findings that (i) not only sleeping problems alarmingly increased across age groups, but also that (ii) daily life habits changed to maladaptive responses such as moving less and spending less time in natural daylight, as well as an increased media device usage.

In conclusion, our data show considerable changes in sleep patterns and behavior during COVID-19 as compared to before the pandemic. Overall, a reduced sleep quality marked by an increase in sleeping problems, irregular bedtimes as well as increased daytime sleepiness and daytime sleep was observed. Furthermore, a stronger COVID-19 related anxiety was correlated with more sleeping problems especially in the youngest (6–10 years). Since sleep disturbances can be an indicator for psychological impairments/ distress, caregivers and clinicians should pay special attention to the behavior of children and adolescents during the pandemic and beyond. Furthermore, sleeping problems during childhood and adolescence can have long-lasting negative effects^90^. Thus, health ministries around the world would be wise to increase their efforts in the implementation of prevention and intervention programs and expand the visibility and accessibility of existing health services for children and adolescents.

## Methods

### Survey design and participants

The online survey^91^ was conducted for a limited time window from February 21, 2021 to April 19, 2021 and targeted 6- to 18-year-old children and adolescents (cross-sectional design). In order to collect nationwide data, the survey was promoted via the Austrian Press Agency (APA), national television (ORF, Austrian Broadcasting Corporation), children and youth organizations, as well as via word of mouth (e.g., WhatsApp groups, Facebook, emails). The recipients of the survey questionnaire link were encouraged to disseminate the invitation to other people.

The survey was divided into two parts: (1) questions about feelings, fears, worries, and thoughts regarding COVID-19 (reported here^17,30^), (2) questions about changes in sleep habits, activity levels and usage of media devices (e.g., smartphone, PC, TV, game console) during COVID-19 and, retrospectively, before the pandemic. After finishing part 1, participants could decide whether they wanted to continue to part 2 or end the survey. Data were organized per age groups: 6–10 years, 11–14 years, and 15–18 years.

Participants entering their email address or phone number at the end of the survey (double chance to win for completing both questionnaire parts) had the chance to win one of 35 vouchers (20€, 30€ or 50€) for tickets from an online ticket platform or a cinema. In agreement with the Ethics Committee of the Austrian Society of Pediatrics and Adolescent Medicine (ÖGKJ) and the legal department of the University of Salzburg, separate informed parental consent was skipped due to the anonymous nature of the survey. Thus, implied consent was obtained when participants completed the (voluntary and anonymous) questionnaire. Furthermore, participants were provided with a link where they could find more detailed information regarding the aim of the survey, privacy policy as well as data processing and results. The survey was approved by the ethics committee of the University of Salzburg (EK-GZ 122013) and conducted in accordance with the Declaration of Helsinki.

Since the time course and extent of COVID-19 related measures is specific to each country, Fig. 8 displays information about the main measures implemented in Austria. During the study period, most parts of Austria were in a reopening phase after about 3.5 months of strict lockdown—except for Vienna, lower Austria and Burgenland which went back into strict lockdown (i.e., “East Lockdown”) between end of March and the end of the study period on April 19th, 2021. During reopening phase, schools operated in a shift system, i.e., classes were divided into two groups: group 1 went to school on Mondays and Tuesdays, group 2 on Wednesdays and Thursdays. On Fridays all students were taught via homeschooling. Leisure activities, e.g., sports clubs, museums, zoos, libraries, private meetings with other families, were possible to a limited degree. Students affected by the “East Lockdown” were taught entirely via homeschooling and were not allowed to leave their homes for non-essential activities.

### Outcome measures

An 18-item investigator-created questionnaire was used to assess changes in sleep habits and behavior of children and adolescents during, and retrospectively, before the COVID-19 pandemic (for the full questionnaire, please refer to Supplementary Information). Participants were asked to indicate how they perceived their sleep and associated behavior as having changed for the better or the worse. In most cases, this was measured as a binary “yes” or “no” item, followed by a rating of the degree to which sleep/behavior had changed (e.g., (1) a lot worse, (2) worse, (3) better, (4) a lot better). However, for some items, the question asked for an immediate rating without a previous “yes”/”no” decision (e.g., (1) a lot worse, (2) worse, (3) no change, (4) better, (5) a lot better).

#### Demographic data

For demographic purposes, gender, age, region of Austria, and school type were assessed (see Supplementary Information Q 1–4).

#### Bed- and wake times

The survey first asked whether participants’ typical bed- and wake times on both weekdays and weekends changed during COVID-19 compared to before. If the answer was “yes” they were asked to indicate in which direction their bed- and wake times changed (e.g., 1 h later) (Q 5–8).

#### General sleep quality, sleeping problems and COVID-19 related anxiety

Furthermore, questions regarding general sleep quality (i.e., change during COVID-19 compared to before; Q 11) and the occurrence of sleeping problems (type, duration and degree of burden) before and during COVID-19 were asked (Q12-17).

To see whether general sleep quality and sleeping problems were reported more frequently in COVID-19 anxious participants, a question from the first part of the survey regarding COVID-19 related anxiety was used (Q 23).

#### Regular bedtimes, time in bed and daytime sleepiness/napping

The survey also asked participants (1) whether there was a change in the regularity of bedtimes during COVID-19 compared to before (Q 10), (2) how their time spent in bed changed during COVID-19 compared to before (Q 9), (3) whether they generally sleep during the day and if so, how often they usually nap (Q 18). Furthermore, nappers were asked whether there were any changes in the frequency of napping during COVID-19 compared to before (Q 19).

#### Physical activity, daylight exposure, usage of media devices

The last part of the survey was devoted to factors that are known to have an impact on sleep and sleeping problems, i.e., (1) physical activity (Q 20), (2) daylight exposure (Q 21), and (3) usage of media devices (Q 22).

### Data analysis

Data was analyzed with jamovi version 1.6.23.0^92^. Descriptive statistics (frequencies, percentages, 95% confidence intervals) were applied to characterize sociodemographic variables, bed- and wake times, general sleep quality, sleeping problems, COVID-19 related anxiety, regular sleep times, time in bed, and daytime sleepiness/napping as well as physical activity, daylight exposure and usage of media devices. Furthermore, nonparametric analyses (Kruskal–Wallis with post-hoc Dwass–Steel–Critchlow–Fligner (DSCF)^93^ test, Mann–Whitney-U test) were conducted to determine differences between age groups as well as between males and females. For changes in the occurrence of sleeping problems before and during COVID-19, Wilcoxon signed-rank tests were calculated. Moreover, effect sizes (rank biserial correlation r, ε^2^) were calculated to indicate the strength of the observed effects^94^. Kendall’s Tau correlation coefficients (*τ*_*b*_) were calculated to test for a relation between COVID-19 related anxiety and (1) general sleep quality and (2) sleeping problems^94–98^. Correlations were designated as small (0.07–0.19), medium (0.20–0.33), and large (> 0.34)^98–102^. For all calculations, p-values less than 0.05 were considered as statistically significant.

## Supplementary Information

Supplementary Information.

## Data Availability

Supplementary Information is provided with the paper to support the current results. Additional data will be shared on reasonable request to the corresponding author.
