# Supplementary material for: Self-reported changes in sleep patterns and behavior in children and adolescents during COVID-19
Source: Sci Rep. 2022 Nov 27;12:20412. doi: 10.1038/s41598-022-24509-7 (PMC9701691; doi:10.1038/s41598-022-24509-7)
Supplement: Supplementary file 1 — Supplementary Information. [file 41598_2022_24509_MOESM1_ESM.pdf]

**SUPPLEMENTARY INFORMATION**

# Self-reported changes in sleep patterns and behavior in children and adolescents during COVID-19

Kathrin Bothe[1], Manuel Schabus[1], Esther-Sevil Eigl[1], Reinhold Kerbl[2], and Kerstin Hoedlmoser[1]

[1] Laboratory for Sleep, Cognition & Consciousness Research, Centre for Cognitive Neuroscience, Department of Psychology, University of Salzburg, Austria

[2] Leoben Hospital, Department of Pediatrics and Adolescent Medicine, Leoben, Austria

**Corresponding author**

Kerstin Hoedlmoser
University of Salzburg, Centre for Cognitive Neuroscience, Laboratory for Sleep, Cognition and Consciousness Research
Hellbrunnerstrasse 34
5020 Salzburg, Austria
Phone.: +43-662-8044-5143
Email: kerstin.hoedlmoser@plus.ac.at

1. You are...

☐ female
☐ male
☐ diverse

2. How old are you?

☐ ☐ years

3. In which state do you live?

☐ Salzburg
☐ Tyrol
☐ Vorarlberg
☐ Carinthia
☐ Lower Austria
☐ Upper Austria
☐ Styria
☐ Vienna
☐ Burgenland

4. What school are you attending?

☐ Elementary school (Volksschule)
☐ Middle school (Neue Mittelschule, AHS Unterstufe, Gymnasium, Sonderschule)
☐ High school (Polytechnische Schule, Berufsschule und Lehre, BMS (Fachschule, Handelsschule), AHS Oberstufe, BHS (HTL, HAK, HLW, BAFEP))

5. Compared to before, do you go to bed at another time on weekdays?

☐ yes ☐ no

↳
☐ more than 2 hours earlier
☐ 2 hours earlier
☐ 1.5 hours earlier
☐ 1 hour earlier
☐ 0.5 hours earlier
☐ 0.5 hours later
☐ 1 hour later
☐ 1.5 hours later
☐ 2 hours later
☐ more than 2 hours later

6. Compared to before, do you go to bed at another time on weekends/holidays/days off?

☐ yes ☐ no

↳
☐ more than 2 hours earlier
☐ 2 hours earlier
☐ 1.5 hours earlier
☐ 1 hour earlier
☐ 0.5 hours earlier
☐ 0.5 hours later
☐ 1 hour later
☐ 1.5 hours later
☐ 2 hours later
☐ more than 2 hours later

7. Compared to before, do you get up at another time on weekdays?

☐ yes ☐ no

↳

| | |
|---|---|
| ☐ more than 2 hours earlier | ☐ 0.5 hours later |
| ☐ 2 hours earlier | ☐ 1 hour later |
| ☐ 1.5 hours earlier | ☐ 1.5 hours later |
| ☐ 1 hour earlier | ☐ 2 hours later |
| ☐ 0.5 hours earlier | ☐ more than 2 hours later |

8. Compared to before, do you get up at another time on weekends/holidays/days off?

yes ☐ no

↳

| | |
|---|---|
| ☐ more than 2 hours earlier | ☐ 0.5 hours later |
| ☐ 2 hours earlier | ☐ 1 hour later |
| ☐ 1.5 hours earlier | ☐ 1.5 hours later |
| ☐ 1 hour earlier | ☐ 2 hours later |
| ☐ 0.5 hours earlier | ☐ more than 2 hours later |

9. How did the time you usually spend in bed change? Please note: time spend in bed includes…
   - the time you are actually asleep
   - the time you need to fall asleep
   - phases of wakefulness during the night, i.e., when you wake up at night, for example, and fall asleep again only after a while
   - the time you spend in bed before sleeping or after waking up, e.g., because it's cosy or you just like lying in bed.

☐ a lot shorter
☐ considerably shorter
☐ slightly shorter
☐ no change
☐ slightly longer
☐ considerably longer
☐ a lot longer

10. Do you go/did you go to bed at the same time every day (+/- 30 min)? This question refers to the time during COVID-19 as well as to the normal state before COVID-19.

☐ yes ☐ no

↳

My bedtime…
☐ is irregular independently of COVID-19
☐ was regular before and got irregular during COVID-19
☐ was irregular before and got regular during COVID-19

11. How would you rate your general sleep quality during COVID-19 compared to before?

☐ a lot worse than before COVID-19
☐ worse than before COVID-19
☐ no change
☐ better than before COVID-19
☐ a lot better than before COVID-19

12. Did you have sleeping problems in the past (before COVID-19)?

☐ yes ☐ no
↳
I …
had problems falling asleep
had problems maintaining sleep
woke up at night for example and had problems falling asleep again
had nightmares and had difficulties falling asleep again
woke up way before the alarm clock and had problems falling asleep again
had problems getting up when the alarm rang
slept excessively
showed unusual behavior, e.g., sleep walking, teeth grinding
showed daytime sleepiness
other

13. How long have you had these sleeping problems?

☐ less than 1 month
☐ 1 month – 6 months
☐ 6 months – 2 years
☐ 2 years – 5 years
☐ more than 5 years

14. How burdensome were these sleeping problems for you?

☐ not at all
☐ slightly
☐ moderately
☐ severely

15. Do you currently have sleeping problems (during COVID-19)?

☐ yes ☐ no
↳
I …
☐ have problems falling asleep
☐ have problems maintaining sleep
☐ wake up at night for example and have problems falling asleep again
☐ have nightmares and have difficulties falling asleep again
☐ wake up way before the alarm clock and have problems falling asleep again
☐ have problems getting up when the alarm rings
☐ sleep excessively

☐ show unusual behavior, e.g., sleep walking, teeth grinding
☐ show daytime sleepiness
☐ other

16. How long have you had these sleeping problems?

☐ less than 1 month
☐ 1 month – 6 months
☐ 6 months – now

17. How burdensome are these sleeping problems for you?

☐ not at all
☐ slightly
☐ moderately
☐ severely

18. Do you usually sleep during the day?

☐ yes ☐ no
↳
I …
☐ nap from time to time
☐ nap regularly
☐ often fall asleep unintentionally during the day

19. Did the frequency of napping change during COVID-19?

☐ yes ☐ no
↳
I …
☐ nap a lot more than before COVID-19
☐ nap more than before COVID-19
☐ nap less than before COVID-19
☐ nap a lot less than before COVID-19

20. Did the extent of physical activity change during COVID-19 compared to before?

☐ yes ☐ no
↳
I am …
☐ a lot less active than before COVID-19
☐ less active than before COVID-19
☐ more active than before COVID-19
☐ a lot more active than before COVID-19

21. How much time do you spend outdoors (including time spent on balcony and in the garden) in daylight during COVID-19 compared to before?

☐ a lot less than before COVID-19
☐ less than before COVID-19
no change
☐ more than before COVID-19
☐ a lot more than before COVID-19

22. Do you spend more time with smartphone/TV/gaming console/tablet/PC etc. during COVID- 19 compared to before?

☐ yes ☐ no

↳

☐ a lot less than before COVID-19
☐ less than before COVID-19
☐ more than before COVID-19
☐ a lot more than before COVID-19

23. Does the current situation with COVID-19 scare you?

yes, a lot
yes, a bit
hardly
not at all

**Table S1.** Bed and wake times on weekdays and weekends/holidays/days off before and during COVID-19.

| | | 6-10 years | | | | | 11-14 years | | | | | 15-18 years | | | | |
|---|---|---|---|---|---|---|---|---|---|---|---|---|---|---|---|---|
| | | | | | 95% CI | | | | | 95% CI | | | | | 95% CI | |
| | **response option** | **n** | **N** | **%** | **Lower** | **Upper** | **n** | **N** | **%** | **Lower** | **Upper** | **n** | **N** | **%** | **Lower** | **Upper** |
| Compared to before, do you go to bed at another time on weekdays? | [1] yes | 150 | 375 | 40.0 | 35.01 | 45.15 | 687 | 952 | 72.2 | 69.20 | 74.99 | 707 | 930 | 76.0 | 73.14 | 78.73 |
| | [2] no | 225 | 375 | 60.0 | 54.85 | 65.00 | 265 | 952 | 27.8 | 25.01 | 30.80 | 223 | 930 | 24.0 | 21.27 | 26.86 |
| | [1] more than 2 hours earlier | 1 | 150 | 0.7 | 0.02 | 3.66 | 9 | 687 | 1.3 | 0.60 | 2.47 | 6 | 707 | 0.8 | 0.31 | 1.84 |
| | [2] 2 hours earlier | 2 | 150 | 1.3 | 0.16 | 4.73 | 5 | 687 | 0.7 | 0.24 | 1.69 | 11 | 707 | 1.6 | 0.78 | 2.77 |
| | [3] 1.5 hours earlier | 2 | 150 | 1.3 | 0.16 | 4.73 | 4 | 687 | 0.6 | 0.16 | 1.48 | 9 | 707 | 1.3 | 0.58 | 2.40 |
| | [4] 1 hour earlier | 1 | 150 | 0.7 | 0.02 | 3.66 | 11 | 687 | 1.6 | 0.80 | 2.85 | 16 | 707 | 2.3 | 1.30 | 3.65 |
| | [5] 0.5 hours earlier | 1 | 150 | 0.7 | 0.02 | 3.66 | 8 | 687 | 1.2 | 0.50 | 2.28 | 5 | 707 | 0.7 | 0.23 | 1.64 |
| | [6] 0.5 hours later | 30 | 150 | 20.0 | 13.92 | 27.30 | 82 | 687 | 11.9 | 9.61 | 14.60 | 21 | 707 | 3.0 | 1.85 | 4.50 |
| | [7] 1 hour later | 60 | 150 | 40.0 | 32.10 | 48.31 | 174 | 687 | 25.3 | 22.12 | 28.75 | 124 | 707 | 17.5 | 14.81 | 20.55 |
| | [8] 1.5 hours later | 27 | 150 | 18.0 | 12.21 | 25.10 | 124 | 687 | 18.0 | 15.24 | 21.13 | 137 | 707 | 19.4 | 16.53 | 22.49 |
| | [9] 2 hours later | 19 | 150 | 12.7 | 7.80 | 19.07 | 112 | 687 | 16.3 | 13.62 | 19.28 | 172 | 707 | 24.3 | 21.21 | 27.66 |
| | [10] more than 2 hours later | 7 | 150 | 4.7 | 1.90 | 9.38 | 158 | 687 | 23.0 | 19.90 | 26.33 | 206 | 707 | 29.1 | 25.81 | 32.64 |
| Compared to before, do you go to bed at another time on weekends/holidays/days off? | [1] yes | 117 | 375 | 31.2 | 26.54 | 36.16 | 411 | 952 | 43.2 | 40.00 | 46.39 | 438 | 930 | 47.1 | 43.85 | 50.36 |
| | [2] no | 258 | 375 | 68.8 | 63.84 | 73.46 | 541 | 952 | 56.8 | 53.61 | 60.00 | 492 | 930 | 52.9 | 49.64 | 56.15 |
| | [1] more than 2 hours earlier | 3 | 117 | 2.6 | 0.53 | 7.31 | 12 | 411 | 2.9 | 1.52 | 5.04 | 38 | 438 | 8.7 | 6.21 | 11.71 |
| | [2] 2 hours earlier | | | | | | 5 | 411 | 1.2 | 0.40 | 2.82 | 19 | 438 | 4.3 | 2.63 | 6.69 |
| | [3] 1.5 hours earlier | | | | | | 6 | 411 | 1.5 | 0.54 | 3.15 | 11 | 438 | 2.5 | 1.26 | 4.45 |
| | [4] 1 hour earlier | 4 | 117 | 3.4 | 0.94 | 8.52 | 7 | 411 | 1.7 | 0.69 | 3.48 | 39 | 438 | 8.9 | 6.41 | 11.97 |
| | [5] 0.5 hours earlier | | | | | | 4 | 411 | 1.0 | 0.27 | 2.47 | 4 | 438 | 0.9 | 0.25 | 2.32 |
| | [6] 0.5 hours later | 9 | 117 | 7.7 | 3.58 | 14.10 | 26 | 411 | 6.3 | 4.17 | 9.13 | 5 | 438 | 1.1 | 0.37 | 2.64 |
| | [7] 1 hour later | 28 | 117 | 23.9 | 16.53 | 32.70 | 65 | 411 | 15.8 | 12.42 | 19.71 | 36 | 438 | 8.2 | 5.82 | 11.20 |
| | [8] 1.5 hours later | 18 | 117 | 15.4 | 9.38 | 23.22 | 43 | 411 | 10.5 | 7.68 | 13.83 | 36 | 438 | 8.2 | 5.82 | 11.20 |
| | [9] 2 hours later | 35 | 117 | 29.9 | 21.80 | 39.07 | 89 | 411 | 21.7 | 17.77 | 25.96 | 71 | 438 | 16.2 | 12.88 | 20.00 |
| | [10] more than 2 hours later | 20 | 117 | 17.1 | 10.77 | 25.16 | 154 | 411 | 37.5 | 32.77 | 42.35 | 179 | 438 | 40.9 | 36.23 | 45.63 |

| | | 6-10 years | | | | | 11-14 years | | | | | 15-18 years | | | | |
|---|---|---|---|---|---|---|---|---|---|---|---|---|---|---|---|---|
| | | | | | 95% CI | | | | | 95% CI | | | | | 95% CI | |
| | response option | n | N | % | Lower | Upper | n | N | % | Lower | Upper | n | N | % | Lower | Upper |
| Compared to before, do you get up at another time on weekdays? | [1] yes | 137 | 375 | 36.5 | 31.65 | 41.63 | 685 | 952 | 72.0 | 68.98 | 74.79 | 754 | 930 | 81.1 | 78.41 | 83.55 |
| | [2] no | 238 | 375 | 63.5 | 58.37 | 68.35 | 267 | 952 | 28.0 | 25.21 | 31.02 | 176 | 930 | 18.9 | 16.46 | 21.59 |
| | [1] more than 2 hours earlier | 1 | 137 | 0.7 | 0.02 | 4.00 | 15 | 685 | 2.2 | 1.23 | 3.59 | 7 | 754 | 0.9 | 0.37 | 1.90 |
| | [2] 2 hours earlier | 1 | 137 | 0.7 | 0.02 | 4.00 | 8 | 685 | 1.2 | 0.51 | 2.29 | 3 | 754 | 0.4 | 0.08 | 1.16 |
| | [3] 1.5 hours earlier | 2 | 137 | 1.5 | 0.18 | 5.17 | 3 | 685 | 0.4 | 0.09 | 1.27 | 12 | 754 | 1.6 | 0.83 | 2.76 |
| | [4] 1 hour earlier | 7 | 137 | 5.1 | 2.08 | 10.24 | 16 | 685 | 2.3 | 1.34 | 3.77 | 26 | 754 | 3.4 | 2.27 | 5.01 |
| | [5] 0.5 hours earlier | 4 | 137 | 2.9 | 0.80 | 7.31 | 31 | 685 | 4.5 | 3.10 | 6.36 | 8 | 754 | 1.1 | 0.46 | 2.08 |
| | [6] 0.5 hours later | 33 | 137 | 24.1 | 17.20 | 32.13 | 175 | 685 | 25.5 | 22.32 | 28.99 | 89 | 754 | 11.8 | 9.59 | 14.32 |
| | [7] 1 hour later | 50 | 137 | 36.5 | 28.44 | 45.15 | 236 | 685 | 34.5 | 30.89 | 38.15 | 248 | 754 | 32.9 | 29.54 | 36.37 |
| | [8] 1.5 hours later | 16 | 137 | 11.7 | 6.83 | 18.27 | 98 | 685 | 14.3 | 11.77 | 17.16 | 144 | 754 | 19.1 | 16.35 | 22.09 |
| | [9] 2 hours later | 18 | 137 | 13.1 | 7.98 | 19.97 | 53 | 685 | 7.7 | 5.85 | 10.00 | 117 | 754 | 15.5 | 13.01 | 18.30 |
| | [10] more than 2 hours later | 5 | 137 | 3.6 | 1.20 | 8.31 | 50 | 685 | 7.3 | 5.47 | 9.51 | 100 | 754 | 13.3 | 10.92 | 15.89 |
| Compared to before, do you get up at another time on weekends/holidays/days off? | [1] yes | 84 | 375 | 22.4 | 18.28 | 26.96 | 331 | 952 | 34.8 | 31.74 | 37.89 | 334 | 930 | 35.9 | 32.83 | 39.09 |
| | [2] no | 291 | 375 | 77.6 | 73.04 | 81.72 | 621 | 952 | 65.2 | 62.11 | 68.26 | 596 | 930 | 64.1 | 60.91 | 67.17 |
| | [1] more than 2 hours earlier | 2 | 84 | 2.4 | 0.29 | 8.34 | 19 | 331 | 5.7 | 3.49 | 8.82 | 18 | 334 | 5.4 | 3.23 | 8.38 |
| | [2] 2 hours earlier | | | | | | 8 | 331 | 2.4 | 1.05 | 4.71 | 15 | 334 | 4.5 | 2.54 | 7.30 |
| | [3] 1.5 hours earlier | 7 | 84 | 8.3 | 3.42 | 16.42 | 10 | 331 | 3.0 | 1.46 | 5.49 | 22 | 334 | 6.6 | 4.17 | 9.80 |
| | [4] 1 hour earlier | 3 | 84 | 3.6 | 0.74 | 10.08 | 16 | 331 | 4.8 | 2.79 | 7.73 | 34 | 334 | 10.2 | 7.15 | 13.93 |
| | [5] 0.5 hours earlier | 5 | 84 | 6.0 | 1.96 | 13.35 | 19 | 331 | 5.7 | 3.49 | 8.82 | 6 | 334 | 1.8 | 0.66 | 3.87 |
| | [6] 0.5 hours later | 12 | 84 | 14.3 | 7.61 | 23.62 | 17 | 331 | 5.1 | 3.02 | 8.10 | 4 | 334 | 1.2 | 0.33 | 3.04 |
| | [7] 1 hour later | 17 | 84 | 20.2 | 12.25 | 30.41 | 57 | 331 | 17.2 | 13.31 | 21.73 | 38 | 334 | 11.4 | 8.18 | 15.28 |
| | [8] 1.5 hours later | 16 | 84 | 19.0 | 11.30 | 29.08 | 41 | 331 | 12.4 | 9.04 | 16.43 | 29 | 334 | 8.7 | 5.89 | 12.23 |
| | [9] 2 hours later | 11 | 84 | 13.1 | 6.72 | 22.22 | 66 | 331 | 19.9 | 15.77 | 24.66 | 56 | 334 | 16.8 | 12.92 | 21.21 |
| | [10] more than 2 hours later | 11 | 84 | 13.1 | 6.72 | 22.22 | 78 | 331 | 23.6 | 19.10 | 28.51 | 112 | 334 | 33.5 | 28.49 | 38.87 |

**Notes:** CI, 95% confidence interval with lower and upper border; empty spaces: response option was not chosen in this age group

**Table S2.** Comparison of bed and wake times across age groups on weekdays and weekends/holidays/days off before and during COVID-19.

| | response option | | age group | n | M | $\chi^2$ | df | p | ε² | DSCF |
|---|---|---|---|---|---|---|---|---|---|---|
| Compared to before, do you go to bed at another time on weekdays? | [1] yes<br>[2] no | all | 6-10 years | 375 | 1.60 | 171.12 | 2 | **< 0.001** | 0.08 | 6-10 > 11-14 , W= -15.45, **p < 0.001** |
| | | | 11-14 years | 952 | 1.28 | | | | | 6-10 > 15-18, W= -17.53, **p < 0.001** |
| | | | 15-18 years | 930 | 1.24 | | | | | 11-14 vs. 15-18, W= -2.70, n.s. |
| | | female | 6-10 years | 187 | 1.62 | 103.39 | 2 | **< 0.001** | 0.07 | 6-10 > 11-14 , W= -11.97, **p < 0.001** |
| | | | 11-14 years | 576 | 1.28 | | | | | 6-10 > 15-18, W= -13.94, **p < 0.001** |
| | | | 15-18 years | 676 | 1.24 | | | | | 11-14 vs. 15-18, W= -2.17, n.s. |
| | | male | 6-10 years | 188 | 1.58 | 65.87 | 2 | **< 0.001** | 0.08 | 6-10 > 11-14 , W= -9.80, **p < 0.001** |
| | | | 11-14 years | 376 | 1.28 | | | | | 6-10 > 15-18, W= -10.25, **p < 0.001** |
| | | | 15-18 years | 254 | 1.24 | | | | | 11-14 vs. 15-18, W= -1.54, n.s. |
| | [1]more than 2 hours earlier<br>[2] 2 hours earlier<br>[3] 1.5 hours earlier<br>[4] 1 hour earlier<br>[5] 0.5 hours earlier<br>[6] 0.5 hours later<br>[7] 1 hour later<br>[8] 1.5 hours later<br>[9] 2 hours later<br>[10] more than 2 hours later | all | 6-10 years | 150 | 7.19 | 87.08 | 2 | **< 0.001** | 0.06 | 6-10 < 11-14 , W= 7.48, **p < 0.001** |
| | | | 11-14 years | 687 | 7.83 | | | | | 6-10 < 15-18, W= 12.71, **p < 0.001** |
| | | | 15-18 years | 707 | 8.32 | | | | | 11-14 < 15-18, W= 7.77, **p < 0.001** |
| | | female | 6-10 years | 71 | 7.38 | 32.45 | 2 | **< 0.001** | 0.03 | 6-10 < 11-14 , W= 5.34, **p = 0.004** |
| | | | 11-14 years | 416 | 8.00 | | | | | 6-10 < 15-18, W= 7.83, **p < 0.001** |
| | | | 15-18 years | 514 | 8.19 | | | | | 11-14 < 15-18, W= 4.05, **p = 0.012** |
| | | male | 6-10 years | 79 | 7.00 | 55.40 | 2 | **< 0.001** | 0.10 | 6-10 < 11-14 , W= 4.57, **p = 0.004** |
| | | | 11-14 years | 271 | 7.66 | | | | | 6-10 < 15-18, W= 10.08, **p < 0.001** |
| | | | 15-18 years | 193 | 8.45 | | | | | 11-14 < 15-18, W= 7.39, **p < 0.001** |
| Compared to before, do you go to bed at another time on weekends/holidays/days off? | [1] yes<br>[2] no | all | 6-10 years | 375 | 1.69 | 27.67 | 2 | **< 0.001** | 0.01 | 6-10 > 11-14 , W= -5.67, **p < 0.001** |
| | | | 11-14 years | 952 | 1.57 | | | | | 6-10 > 15-18, W= -7.43, **p < 0.001** |
| | | | 15-18 years | 930 | 1.55 | | | | | 11-14 vs. 15-18, W= -2.42, n.s. |
| | | female | 6-10 years | 187 | 1.69 | 20.42 | 2 | **< 0.001** | 0.01 | 6-10 > 11-14 , W= -4.35, **p = 0.006** |
| | | | 11-14 years | 576 | 1.56 | | | | | 6-10 > 15-18, W= -6.32, **p < 0.001** |
| | | | 15-18 years | 676 | 1.51 | | | | | 11-14 vs. 15-18, W= -2.83, n.s. |
| | | male | 6-10 years | 188 | 1.69 | 6.62 | 2 | **0.036** | 0.01 | 6-10 > 11-14 , W= -3.54, **p = 0.033** |
| | | | 11-14 years | 376 | 1.58 | | | | | 6-10 vs. 15-18, W= -2.91, n.s. |
| | | | 15-18 years | 254 | 1.59 | | | | | 11-14 vs. 15-18, W= 0.47, n.s. |
| | [1]more than 2 hours earlier<br>[2] 2 hours earlier<br>[3] 1.5 hours earlier<br>[4] 1 hour earlier<br>[5] 0.5 hours earlier<br>[6] 0.5 hours later<br>[7] 1 hour later<br>[8] 1.5 hours later<br>[9] 2 hours later<br>[10] more than 2 hours later | all | 6-10 years | 117 | 7.93 | 7.86 | 2 | **0.020** | 0.01 | 6-10 < 11-14 , W=4.15, **p = 0.009** |
| | | | 11-14 years | 411 | 8.19 | | | | | 6-10 vs. 15-18, W= 2.45, n.s. |
| | | | 15-18 years | 438 | 7.79 | | | | | 11-14 > 15-18, W= -1.81, n.s. |
| | | female | 6-10 years | 58 | 8.03 | 12.10 | 2 | **0.002** | 0.02 | 6-10 < 11-14 , W=3.67, **p = 0.026** |
| | | | 11-14 years | 252 | 8.40 | | | | | 6-10 vs. 15-18, W= 0.43, n.s. |
| | | | 15-18 years | 334 | 7.24 | | | | | 11-14 > 15-18, W= -4.44, **p =0.005** |
| | | male | 6-10 years | 59 | 7.83 | 13.96 | 2 | **< 0.001** | 0.04 | 6-10 vs. 11-14 , W=1.71, n.s. |
| | | | 11-14 years | 159 | 7.97 | | | | | 6-10 < 15-18, W= 4.97, **p = 0.001** |
| | | | 15-18 years | 104 | 8.34 | | | | | 11-14 < 15-18, W= 4.10, **p =0.011** |

| | response option | | age group | n | M | $\chi^2$ | df | p | ε² | DSCF |
|---|---|---|---|---|---|---|---|---|---|---|
| Compared to before, do you get up at another time on weekdays? | [1] yes<br>[2] no | | 6-10 years | 375 | 1.64 | | | | | 6-10 > 11-14 , W= -16.92, **p < 0.001** |
| | | all | 11-14 years | 952 | 1.28 | 255.07 | 2 | **< 0.001** | 0.11 | 6-10 > 15-18, W= -22.12, **p < 0.001** |
| | | | 15-18 years | 930 | 1.21 | | | | | 11-14 > 15-18, W= -6.59, **p < 0.001** |
| | | | 6-10 years | 187 | 1.64 | | | | | 6-10 > 11-14 , W= -12.15, **p < 0.001** |
| | | female | 11-14 years | 576 | 1.29 | 167.31 | 2 | **< 0.001** | 0.12 | 6-10 > 15-18, W= -18.43, **p < 0.001** |
| | | | 15-18 years | 676 | 1.16 | | | | | 11-14 > 15-18, W= -7.55, **p < 0.001** |
| | | | 6-10 years | 188 | 1.63 | | | | | 6-10 > 11-14 , W= -11.72, **p < 0.001** |
| | | male | 11-14 years | 376 | 1.27 | 84.37 | 2 | **< 0.001** | 0.10 | 6-10 > 15-18, W= -10.98, **p < 0.001** |
| | | | 15-18 years | 254 | 1.26 | | | | | 11-14 vs. 15-18, W= -0.29, n.s. |
| | [1]more than 2 hours earlier<br>[2] 2 hours earlier<br>[3] 1.5 hours earlier<br>[4] 1 hour earlier<br>[5] 0.5 hours earlier<br>[6] 0.5 hours later<br>[7] 1 hour later<br>[8] 1.5 hours later<br>[9] 2 hours later<br>[10] more than 2 hours later | | 6-10 years | 137 | 6.90 | | | | | 6-10 vs. 11-14 , W= 8.63, n.s. |
| | | all | 11-14 years | 685 | 6.88 | 73.31 | 2 | **< 0.001** | 0.05 | 6-10 < 15-18, W= 6.66, **p < 0.001** |
| | | | 15-18 years | 754 | 7.54 | | | | | 11-14 < 15-18, W= 11.53, **p < 0.001** |
| | | | 6-10 years | 68 | 6.97 | | | | | 6-10 vs. 11-14 , W= -0.25, n.s. |
| | | female | 11-14 years | 411 | 6.93 | 42.26 | 2 | **< 0.001** | 0.04 | 6-10 < 15-18, W= 4.70, **p = 0.003** |
| | | | 15-18 years | 567 | 7.50 | | | | | 11-14 < 15-18, W= 8.71, **p < 0.001** |
| | | | 6-10 years | 69 | 6.83 | | | | | 6-10 vs. 11-14 , W= 0.16, n.s. |
| | | male | 11-14 years | 274 | 6.83 | 26.87 | 2 | **< 0.001** | 0.05 | 6-10 < 15-18, W= 4.58, **p = 0.003** |
| | | | 15-18 years | 187 | 7.57 | | | | | 11-14 < 15-18, W= 7.08, **p < 0.001** |
| Compared to before, do you get up at another time on weekends/holidays/days off? | [1] yes<br>[2] no | | 6-10 years | 375 | 1.78 | | | | | 6-10 > 11-14 , W=-6.19, **p < 0.001** |
| | | all | 11-14 years | 952 | 1.66 | 23.86 | 2 | **< 0.001** | 0.01 | 6-10 > 15-18, W= -6.69, **p < 0.001** |
| | | | 15-18 years | 930 | 1.64 | | | | | 11-14 vs. 15-18, W= -0.74, n.s. |
| | | | 6-10 years | 187 | 1.76 | | | | | 6-10 > 11-14 , W=-4.47, **p = 0.005** |
| | | female | 11-14 years | 576 | 1.63 | 10.65 | 2 | **0.005** | 0.01 | 6-10 > 15-18, W= -4.26, **p = 0.007** |
| | | | 15-18 years | 676 | 1.64 | | | | | 11-14 vs. 15-18, W= 0.43, n.s. |
| | | | 6-10 years | 188 | 1.79 | | | | | 6-10 > 11-14 , W=-3.93, **p = 0.015** |
| | | male | 11-14 years | 376 | 1.68 | 12.65 | 2 | **0.002** | 0.02 | 6-10 > 15-18, W= -4.98, **p = 0.001** |
| | | | 15-18 years | 254 | 1.64 | | | | | 11-14 vs. 15-18, W= - 1.59, n.s. |
| | [1]more than 2 hours earlier<br>[2] 2 hours earlier<br>[3] 1.5 hours earlier<br>[4] 1 hour earlier<br>[5] 0.5 hours earlier<br>[6] 0.5 hours later<br>[7] 1 hour later<br>[8] 1.5 hours later<br>[9] 2 hours later<br>[10] more than 2 hours later | | 6-10 years | 84 | 7.00 | | | | | 6-10 vs. 11-14 , W= 2.85, n.s. |
| | | all | 11-14 years | 331 | 7.32 | 6.10 | 2 | **0.047** | 0.01 | 6-10 vs. 15-18, W= 3.26, n.s. |
| | | | 15-18 years | 334 | 7.39 | | | | | 11-14 vs. 15-18, W= 1.45, n.s. |
| | | | 6-10 years | 45 | 7.02 | | | | | 6-10 vs. 11-14 , W= 2.59, n.s. |
| | | female | 11-14 years | 211 | 7.37 | 3.01 | 2 | 0.222 | 0.01 | 6-10 vs. 15-18, W= 2.07, n.s. |
| | | | 15-18 years | 242 | 7.12 | | | | | 11-14 vs. 15-18, W= -0.37, n.s. |
| | | | 6-10 years | 39 | 6.97 | | | | | 6-10 vs. 11-14 , W= 1.43, n.s. |
| | | male | 11-14 years | 120 | 7.26 | 8.22 | 2 | **0.016** | 0.03 | 6-10 vs. 15-18, W= 3.29, n.s. |
| | | | 15-18 years | 92 | 7.65 | | | | | 11-14 < 15-18, W= 3.39, **p = 0.044** |

Notes: effect size ε²: < 0.04 very small to small effect; 0.04-0.16 medium effect; 0.16-0.64 large effect; > 0.64 very large effect; DSCF: Dwass-Steel-Critchlow-Fligner test corrects for multiple comparisons.

**Table S3**. Comparison of bed and wake times in males and females on weekdays and weekends/holidays/days off before and during COVID-19.

| | response option | age group | N | M | sex | n | M | U | p | r |
|---|---|---|---|---|---|---|---|---|---|---|
| Compared to before, do you go to bed at another time on weekdays? | [1] yes<br>[2] no | 6-10 years | 375 | 1.60 | female | 187 | 1.62 | 16865.5 | 0.424 | 0.04 |
| | | | | | male | 188 | 1.58 | | | |
| | | 11-14 years | 952 | 1.28 | female | 576 | 1.28 | 108128.0 | 0.960 | 0.00 |
| | | | | | male | 376 | 1.28 | | | |
| | | 15-18 years | 930 | 1.24 | female | 676 | 1.24 | 85808.0 | 0.987 | 0.00 |
| | | | | | male | 254 | 1.24 | | | |
| | | 6-18 years | 2257 | 1.32 | female | 1439 | 1.30 | 569831.0 | 0.118 | 0.03 |
| | | | | | male | 818 | 1.34 | | | |
| | [1] more than 2 hours earlier<br>[2] 2 hours earlier<br>[3] 1.5 hours earlier<br>[4] 1 hour earlier<br>[5] 0.5 hours earlier<br>[6] 0.5 hours later<br>[7] 1 hour later<br>[8] 1.5 hours later<br>[9] 2 hours later<br>[10] more than 2 hours later | 6-10 years | 150 | 7.19 | female | 71 | 7.38 | 2501.0 | 0.234 | 0.11 |
| | | | | | male | 79 | 7.00 | | | |
| | | 11-14 years | 687 | 7.83 | female | 416 | 8.00 | 49974.0 | **0.010** | 0.11 |
| | | | | | male | 271 | 7.66 | | | |
| | | 15-18 years | 707 | 8.32 | female | 514 | 8.19 | 46683.0 | 0.216 | 0.06 |
| | | | | | male | 193 | 8.45 | | | |
| | | 6-18 years | 1544 | 7.95 | female | 1001 | 8.06 | 248564.0 | **0.005** | 0.09 |
| | | | | | male | 543 | 7.84 | | | |
| Compared to before, do you go to bed at another time on weekends/holidays/days off? | [1] yes<br>[2] no | 6-10 years | 375 | 1.69 | female | 187 | 1.69 | 17513.5 | 0.939 | 0.00 |
| | | | | | male | 188 | 1.69 | | | |
| | | 11-14 years | 952 | 1.57 | female | 576 | 1.56 | 106704.0 | 0.656 | 0.01 |
| | | | | | male | 376 | 1.58 | | | |
| | | 15-18 years | 930 | 1.55 | female | 676 | 1.51 | 78586.0 | **0.021** | 0.08 |
| | | | | | male | 254 | 1.59 | | | |
| | | 6-18 years | 2257 | 1.58 | female | 1439 | 1.55 | 556834.0 | **0.013** | 0.05 |
| | | | | | male | 818 | 1.61 | | | |
| | [1] more than 2 hours earlier<br>[2] 2 hours earlier<br>[3] 1.5 hours earlier<br>[4] 1 hour earlier<br>[5] 0.5 hours earlier<br>[6] 0.5 hours later<br>[7] 1 hour later<br>[8] 1.5 hours later<br>[9] 2 hours later<br>[10] more than 2 hours later | 6-10 years | 117 | 7.93 | female | 58 | 8.03 | 1608.5 | 0.568 | 0.06 |
| | | | | | male | 59 | 7.83 | | | |
| | | 11-14 years | 411 | 8.19 | female | 252 | 8.40 | 17354.0 | **0.018** | 0.13 |
| | | | | | male | 159 | 7.97 | | | |
| | | 15-18 years | 438 | 7.79 | female | 334 | 7.24 | 14082.0 | **0.002** | 0.19 |
| | | | | | male | 104 | 8.34 | | | |
| | | 6-18 years | 966 | 7.91 | female | 644 | 7.76 | 102413.0 | 0.748 | 0.01 |
| | | | | | male | 322 | 8.06 | | | |

| | response option | age group | N | M | sex | n | M | U | p | r |
|---|---|---|---|---|---|---|---|---|---|---|
| Compared to before, do you get up at another time on weekdays? | [1] yes<br>[2] no | 6-10 years | 375 | 1.64 | female<br>male | 187<br>188 | 1.64<br>1.63 | 17518.5 | 0.946 | 0.00 |
| | | 11-14 years | 952 | 1.28 | female<br>male | 576<br>376 | 1.29<br>1.27 | 106644.0 | 0.611 | 0.02 |
| | | 15-18 years | 930 | 1.21 | female<br>male | 676<br>254 | 1.16<br>1.26 | 77049.0 | **< 0.001** | 0.10 |
| | | 6-18 years | 2257 | 1.31 | female<br>male | 1439<br>818 | 1.27<br>1.35 | 542072.0 | **< 0.001** | 0.08 |
| | [1] more than 2 hours earlier<br>[2] 2 hours earlier<br>[3] 1.5 hours earlier<br>[4] 1 hour earlier<br>[5] 0.5 hours earlier<br>[6] 0.5 hours later<br>[7] 1 hour later<br>[8] 1.5 hours later<br>[9] 2 hours later<br>[10] more than 2 hours later | 6-10 years | 137 | 6.90 | female<br>male | 68<br>69 | 6.97<br>6.83 | 2221.0 | 0.579 | 0.05 |
| | | 11-14 years | 685 | 6.88 | female<br>male | 411<br>274 | 6.93<br>6.83 | 54316.0 | 0.418 | 0.04 |
| | | 15-18 years | 754 | 7.54 | female<br>male | 567<br>187 | 7.50<br>7.57 | 51917.0 | 0.663 | 0.02 |
| | | 6-18 years | 1576 | 7.17 | female<br>male | 1046<br>530 | 7.24<br>7.09 | 259849.0 | **0.037** | 0.06 |
| Compared to before, do you get up at another time on weekends/holidays/days off? | [1] yes<br>[2] no | 6-10 years | 375 | 1.78 | female<br>male | 187<br>188 | 1.76<br>1.79 | 16994.5 | 0.442 | 0.03 |
| | | 11-14 years | 952 | 1.66 | female<br>male | 576<br>376 | 1.63<br>1.68 | 103180.0 | 0.135 | 0.05 |
| | | 15-18 years | 930 | 1.64 | female<br>male | 676<br>254 | 1.64<br>1.64 | 85490.0 | 0.905 | 0.00 |
| | | 6-18 years | 2257 | 1.67 | female<br>male | 1439<br>818 | 1.65<br>1.69 | 565464.0 | 0.057 | 0.04 |
| | [1] more than 2 hours earlier<br>[2] 2 hours earlier<br>[3] 1.5 hours earlier<br>[4] 1 hour earlier<br>[5] 0.5 hours earlier<br>[6] 0.5 hours later<br>[7] 1 hour later<br>[8] 1.5 hours later<br>[9] 2 hours later<br>[10] more than 2 hours later | 6-10 years | 84 | 7.00 | female<br>male | 45<br>39 | 7.02<br>6.97 | 863.5 | 0.902 | 0.02 |
| | | 11-14 years | 331 | 7.32 | female<br>male | 211<br>120 | 7.37<br>7.26 | 11891.0 | 0.352 | 0.06 |
| | | 15-18 years | 334 | 7.39 | female<br>male | 242<br>92 | 7.12<br>7.65 | 9764.0 | 0.076 | 0.12 |
| | | 6-18 years | 749 | 7.29 | female<br>male | 498<br>251 | 7.22<br>7.36 | 61376.0 | 0.683 | 0.02 |

**Notes:** effect size r = biserial rank correlation: < 0.28 very small to small effect; 0.28 – 0.43 medium effect; > 0.43 large effect

**Table S4.** Changes in general sleep quality and sleeping problems.

| | | 6-10 years | | | | | 11-14 years | | | | | 15-18 years | | | | |
|---|---|---|---|---|---|---|---|---|---|---|---|---|---|---|---|---|
| | | | | | 95% CI | | | | | 95% CI | | | | | 95% CI | |
| | response option | n | N | % | Lower | Upper | n | N | % | Lower | Upper | n | N | % | Lower | Upper |
| How would you rate your general sleep quality during COVID-19 compared to before? | [1] a lot worse than before COVID-19 | 15 | 375 | 4.0 | 2.26 | 6.51 | 84 | 951 | 8.8 | 7.11 | 10.82 | 92 | 930 | 9.9 | 8.05 | 11.99 |
| | [2] worse than before COVID-19 | 107 | 375 | 28.5 | 24.02 | 33.39 | 249 | 951 | 26.2 | 23.41 | 29.10 | 290 | 930 | 31.2 | 28.22 | 34.27 |
| | [3] no change | 235 | 375 | 62.7 | 57.55 | 67.58 | 528 | 951 | 55.5 | 52.30 | 58.71 | 458 | 930 | 49.2 | 45.99 | 52.51 |
| | [4] better than before COVID-19 | 17 | 375 | 4.5 | 2.66 | 7.16 | 68 | 951 | 7.2 | 5.60 | 8.98 | 75 | 930 | 8.1 | 6.40 | 10.00 |
| | [5] a lot better than before COVID-19 | 1 | 375 | 0.3 | 0.01 | 1.48 | 22 | 951 | 2.3 | 1.46 | 3.48 | 15 | 930 | 1.6 | 0.91 | 2.65 |
| Did you have sleeping problems in the past (before COVID-19)? | [1] yes | 50 | 375 | 13.3 | 10.06 | 17.20 | 193 | 952 | 20.3 | 17.76 | 22.97 | 262 | 930 | 28.2 | 25.30 | 31.18 |
| | [2] no | 325 | 375 | 86.7 | 82.80 | 89.94 | 759 | 952 | 79.7 | 77.03 | 82.24 | 668 | 930 | 71.8 | 68.82 | 74.70 |
| | [1] I had problems falling asleep | 26 | 50 | 52.0 | 37.42 | 66.34 | 108 | 193 | 56.0 | 48.65 | 63.08 | 135 | 262 | 51.5 | 45.30 | 57.72 |
| | [2] I had problems maintaining sleep | 10 | 50 | 20.0 | 10.03 | 33.72 | 31 | 193 | 16.1 | 11.18 | 22.02 | 38 | 262 | 14.5 | 10.47 | 19.36 |
| | [3] I had problems getting up when the alarm rang | | | | | | 14 | 193 | 7.3 | 4.02 | 11.87 | 28 | 262 | 10.7 | 7.22 | 15.07 |
| | [4] I had nightmares and had difficulties falling asleep again | 9 | 50 | 18.0 | 8.58 | 31.44 | 10 | 193 | 5.2 | 2.51 | 9.32 | 11 | 262 | 4.2 | 2.11 | 7.39 |
| | [5] I showed daytime sleepiness | 1 | 50 | 2.0 | 0.05 | 10.65 | 5 | 193 | 2.6 | 0.85 | 5.94 | 22 | 262 | 8.4 | 5.34 | 12.44 |
| | [6] I slept excessively | | | | | | 2 | 193 | 1.0 | 0.13 | 3.69 | 3 | 262 | 1.1 | 0.24 | 3.31 |
| | [7] I showed unusual behavior, e.g., sleep walking, teeth grinding | | | | | | 3 | 193 | 1.6 | 0.32 | 4.48 | 2 | 262 | 0.8 | 0.09 | 2.73 |
| | [8] I woke up way before the alarm clock and had problems falling asleep again | | | | | | 3 | 193 | 1.6 | 0.32 | 4.48 | 5 | 262 | 1.9 | 0.62 | 4.40 |
| | [9] other | 4 | 50 | 8.0 | 2.22 | 19.23 | 17 | 193 | 8.8 | 5.22 | 13.73 | 18 | 262 | 6.9 | 4.12 | 10.64 |
| How long have you had these sleeping problems? | [1] less than 1 month | 6 | 50 | 12.0 | 4.53 | 24.31 | 22 | 193 | 11.4 | 7.28 | 16.75 | 21 | 262 | 8.0 | 5.03 | 11.99 |
| | [2] 1 month - 6 months | 13 | 50 | 26.0 | 14.63 | 40.34 | 50 | 193 | 25.9 | 19.88 | 32.69 | 67 | 262 | 25.6 | 20.40 | 31.31 |
| | [3] 6 months - 2 years | 17 | 50 | 34.0 | 21.21 | 48.77 | 49 | 193 | 25.4 | 19.41 | 32.14 | 99 | 262 | 37.8 | 31.89 | 43.96 |
| | [4] 2 years - 5 years | 5 | 50 | 10.0 | 3.33 | 21.81 | 42 | 193 | 21.8 | 16.16 | 28.26 | 41 | 262 | 15.6 | 11.47 | 20.63 |
| | [5] more than 5 years | 9 | 50 | 18.0 | 8.58 | 31.44 | 30 | 193 | 15.5 | 10.74 | 21.44 | 34 | 262 | 13.0 | 9.16 | 17.66 |
| How burdensome were these sleeping problems for you? | [1] not at all | 5 | 50 | 10.0 | 3.33 | 21.81 | 21 | 193 | 10.9 | 6.86 | 16.15 | 18 | 262 | 6.9 | 4.12 | 10.64 |
| | [2] a little | 24 | 50 | 48.0 | 33.66 | 62.58 | 67 | 193 | 34.7 | 28.02 | 41.89 | 81 | 262 | 30.9 | 25.37 | 36.90 |
| | [3] moderately | 11 | 50 | 22.0 | 11.53 | 35.96 | 60 | 193 | 31.1 | 24.64 | 38.13 | 124 | 262 | 47.3 | 41.15 | 53.57 |
| | [4] severely | 10 | 50 | 20.0 | 10.03 | 33.72 | 45 | 193 | 23.3 | 17.54 | 29.93 | 39 | 262 | 14.9 | 10.80 | 19.78 |
| Do you currently have sleeping problems (during COVID-19)? | [1] yes | 124 | 375 | 33.1 | 28.32 | 38.08 | 332 | 952 | 34.9 | 31.85 | 38.00 | 421 | 930 | 45.3 | 42.04 | 48.53 |
| | [2] no | 251 | 375 | 66.9 | 61.92 | 71.68 | 620 | 952 | 65.1 | 62.00 | 68.16 | 509 | 930 | 54.7 | 51.47 | 57.97 |
| | [1] I have problems falling asleep | 50 | 124 | 40.3 | 31.61 | 49.51 | 147 | 332 | 44.3 | 38.86 | 49.80 | 138 | 421 | 32.8 | 28.31 | 37.49 |
| | [2] I have problems maintaining sleep | 25 | 124 | 20.2 | 13.49 | 28.31 | 65 | 332 | 19.6 | 15.45 | 24.26 | 79 | 421 | 18.8 | 15.15 | 22.83 |
| | [3] I have problems getting up when the alarm rings | 4 | 124 | 3.2 | 0.89 | 8.05 | 14 | 332 | 4.2 | 2.32 | 6.97 | 41 | 421 | 9.7 | 7.08 | 12.98 |
| | [4] I have nightmares and have difficulties falling asleep again | 17 | 124 | 13.7 | 8.19 | 21.04 | 30 | 332 | 9.0 | 6.18 | 12.65 | 28 | 421 | 6.7 | 4.47 | 9.47 |
| | [5] I show daytime sleepines | 7 | 124 | 5.6 | 2.30 | 11.29 | 23 | 332 | 6.9 | 4.44 | 10.21 | 59 | 421 | 14.0 | 10.84 | 17.70 |
| | [6] I sleep excessively | 2 | 124 | 1.6 | 0.20 | 5.70 | 7 | 332 | 2.1 | 0.85 | 4.30 | 15 | 421 | 3.6 | 2.01 | 5.81 |
| | [7] I show unusual behavior, e.g., sleep walking, teeth grinding | 3 | 124 | 2.4 | 0.50 | 6.91 | 5 | 332 | 1.5 | 0.49 | 3.48 | 5 | 421 | 1.2 | 0.39 | 2.75 |
| | [8] I wake up way before the alarm clock and have problems falling asleep again | 4 | 124 | 3.2 | 0.89 | 8.05 | 10 | 332 | 3.0 | 1.45 | 5.47 | 18 | 421 | 4.3 | 2.55 | 6.67 |
| | [9] other | 12 | 124 | 9.7 | 5.10 | 16.29 | 31 | 332 | 9.3 | 6.43 | 12.99 | 38 | 421 | 9.0 | 6.47 | 12.18 |

| | response option | 6-10 years | | | | | 11-14 years | | | | | 15-18 years | | | | |
|---|---|---|---|---|---|---|---|---|---|---|---|---|---|---|---|---|
| | | | | | 95% CI | | | | | 95% CI | | | | | 95% CI | |
| | | n | N | % | Lower | Upper | n | N | % | Lower | Upper | n | N | % | Lower | Upper |
| How long have you had these sleeping problems? | [1] less than 1 month | 9 | 124 | 7.3 | 3.37 | 13.33 | 56 | 331 | 16.9 | 13.04 | 21.40 | 54 | 421 | 12.8 | 9.79 | 16.40 |
| | [2] 1 month - 6 months | 61 | 124 | 49.2 | 40.11 | 58.32 | 115 | 331 | 34.7 | 29.62 | 40.14 | 183 | 421 | 43.5 | 38.67 | 48.35 |
| | [3] 6 months - now | 54 | 124 | 43.5 | 34.67 | 52.74 | 160 | 331 | 48.3 | 42.84 | 53.87 | 184 | 421 | 43.7 | 38.91 | 48.59 |
| How burdensome are these sleeping problems for you? | [1] not at all | 8 | 124 | 6.5 | 2.83 | 12.32 | 30 | 332 | 9.0 | 6.18 | 12.65 | 19 | 421 | 4.5 | 2.74 | 6.96 |
| | [2] a little | 48 | 124 | 38.7 | 30.10 | 47.87 | 112 | 332 | 33.7 | 28.66 | 39.10 | 121 | 421 | 28.7 | 24.46 | 33.32 |
| | [3] moderately | 49 | 124 | 39.5 | 30.86 | 48.69 | 117 | 332 | 35.2 | 30.10 | 40.64 | 202 | 421 | 48.0 | 43.12 | 52.87 |
| | [4] severely | 19 | 124 | 15.3 | 9.49 | 22.89 | 73 | 332 | 22.0 | 17.65 | 26.83 | 79 | 421 | 18.8 | 15.15 | 22.83 |

**Notes:** CI, 95% confidence interval with lower and upper border; empty spaces: response option was not chosen in this age group

**Table S5**. Comparison between age groups for changes in type, duration and burden of sleeping problems before and during COVID-19 as well as COVID-19 related anxiety.

| | response option | | age group | n | M | $\chi^2$ | df | p | ε² | DSCF |
|---|---|---|---|---|---|---|---|---|---|---|
| How would you rate your general sleep quality during COVID-19 compared to before? | [1] a lot worse than before COVID-19<br>[2] worse than before COVID-19<br>[3] no change<br>[4] better than before COVID-19<br>[5] a lot better than before COVID-19 | all | 6-10 years | 375 | 2.69 | 6.29 | 2 | **0.043** | 0.00 | 6-10 vs. 11-14 , W= -0.27, n.s. |
| | | | 11-14 years | 952 | 2.70 | | | | | 6-10 vs. 15-18, W= -2.81, n.s. |
| | | | 15-18 years | 930 | 2.63 | | | | | 11-14 vs. 15-18, W= -3.06, n.s. |
| | | female | 6-10 years | 187 | 2.64 | 2.37 | 2 | 0.305 | 0.00 | 6-10 > 11-14 , W=-0.33, **p = 0.010** |
| | | | 11-14 years | 576 | 2.63 | | | | | 6-10 > 15-18, W= -0.75, **p = 0.002** |
| | | | 15-18 years | 676 | 2.57 | | | | | 11-14 vs. 15-18, W= - 1.80, n.s. |
| | | male | 6-10 years | 188 | 2.73 | 1.63 | 2 | 0.443 | 0.00 | 6-10 vs. 11-14 , W= 0.49, n.s. |
| | | | 11-14 years | 376 | 2.76 | | | | | 6-10 vs. 15-18, W= -1.12, n.s. |
| | | | 15-18 years | 254 | 2.69 | | | | | 11-14 vs. 15-18, W= -1.75, n.s. |
| Did you have sleeping problems in the past (before COVID-19)? | [1] yes<br>[2] no | all | 6-10 years | 375 | 1.87 | 38.05 | 2 | **< 0.001** | 0.02 | 6-10 > 11-14 , W=-4.16, **p = 0.009** |
| | | | 11-14 years | 952 | 1.81 | | | | | 6-10 > 15-18, W= -8.04, **p < 0.001** |
| | | | 15-18 years | 930 | 1.75 | | | | | 11-14 > 15-18, W= -5.66, **p < 0.001** |
| | | female | 6-10 years | 187 | 1.84 | 22.16 | 2 | **< 0.001** | 0.02 | 6-10 vs. 11-14 , W=-3.24, n.s. |
| | | | 11-14 years | 576 | 1.77 | | | | | 6-10 > 15-18, W= -6.00, **p < 0.001** |
| | | | 15-18 years | 676 | 1.69 | | | | | 11-14 > 15-18, W= -4.33, **p = 0.006** |
| | | male | 6-10 years | 188 | 1.89 | 6.52 | 2 | **0.038** | 0.01 | 6-10 vs. 11-14 , W= -1.94, n.s. |
| | | | 11-14 years | 376 | 1.85 | | | | | 6-10 > 15-18, W= -3.54, **p = 0.033** |
| | | | 15-18 years | 254 | 1.80 | | | | | 11-14 vs. 15-18, W= -2.14, n.s. |
| Type of sleeping problem (before COVID-19)? | [1] I had problems falling asleep<br>[2] I had problems maintaining sleep<br>[3] I woke up at night, for example, and had problems falling asleep again<br>[4] I had nightmares and had difficulties falling asleep again<br>[5] I woke up way before the alarm clock and had problems falling asleep again<br>[6] I had problems getting up when the alarm rang<br>[7] I slept excessively<br>[8] I showed unusual behavior, e.g., sleep walking, teeth grinding<br>[9] I showed daytime sleepiness<br>[10] other | all | 6-10 years | 50 | 2.45 | 0.93 | 2 | 0.629 | 0.00 | 6-10 vs. 11-14 , W= -0.49, n.s. |
| | | | 11-14 years | 193 | 2.34 | | | | | 6-10 vs. 15-18, W= 0.42, n.s. |
| | | | 15-18 years | 262 | 2.61 | | | | | 11-14 vs. 15-18, W= 1.35, n.s. |
| | | female | 6-10 years | 29 | 2.52 | 0.16 | 2 | 0.926 | 0.00 | 6-10 vs. 11-14 , W= -0.56, n.s. |
| | | | 11-14 years | 135 | 2.81 | | | | | 6-10 vs. 15-18, W= -0.51, n.s. |
| | | | 15-18 years | 211 | 2.61 | | | | | 11-14 vs. 15-18, W= -0.01, n.s. |
| | | male | 6-10 years | 21 | 2.38 | 2.22 | 2 | 0.329 | 0.02 | 6-10 vs. 11-14 , W= -0.38, n.s. |
| | | | 11-14 years | 58 | 1.86 | | | | | 6-10 vs. 15-18, W= 1.11, n.s. |
| | | | 15-18 years | 51 | 2.61 | | | | | 11-14 vs. 15-18, W= 2.07, n.s. |
| How long have you had these sleeping problems? | [1] less than 1 month<br>[2] 1 month - 6 months<br>[3] 6 months - 2 years<br>[4] 2 years - 5 years<br>[5] more than 5 years | all | 6-10 years | 50 | 2.98 | 0.29 | 2 | 0.866 | 0.00 | 6-10 vs. 11-14 , W= 0.64, n.s. |
| | | | 11-14 years | 193 | 3.04 | | | | | 6-10 vs. 15-18, W= 0.50, n.s. |
| | | | 15-18 years | 262 | 3.07 | | | | | 11-14 vs. 15-18, W= -0.50, n.s. |
| | | female | 6-10 years | 29 | 2.86 | 0.96 | 2 | 0.620 | 0.00 | 6-10 vs. 11-14 , W= 1.14, n.s. |
| | | | 11-14 years | 135 | 3.04 | | | | | 6-10 vs. 15-18, W= 0.77, n.s. |
| | | | 15-18 years | 211 | 2.96 | | | | | 11-14 vs. 15-18, W= -1.02, n.s. |
| | | male | 6-10 years | 21 | 3.10 | 0.36 | 2 | 0.834 | 0.00 | 6-10 vs. 11-14 , W= -0.27, n.s. |
| | | | 11-14 years | 58 | 3.03 | | | | | 6-10 vs. 15-18, W= 0.50 n.s. |
| | | | 15-18 years | 51 | 3.18 | | | | | 11-14 vs. 15-18, W= 0.81, n.s. |
| How burdensome were these sleeping problems? | [1] not at all<br>[2] slightly<br>[3] moderately<br>[4] severely | all | 6-10 years | 50 | 2.50 | 2.42 | 2 | 0.299 | 0.00 | 6-10 vs .11-14 , W= 1.55, n.s. |
| | | | 11-14 years | 193 | 2.64 | | | | | 6-10 vs. 15-18, W= 2.34, n.s. |
| | | | 15-18 years | 262 | 2.65 | | | | | 11-14 vs. 15-18, W= 0.65, n.s. |
| | | female | 6-10 years | 29 | 2.62 | 0.64 | 2 | 0.726 | 0.00 | 6-10 vs. 11-14 , W= 0.76, n.s. |
| | | | 11-14 years | 135 | 2.71 | | | | | 6-10 vs 15-18, W= -1.22, n.s. |
| | | | 15-18 years | 211 | 2.74 | | | | | 11-14 vs 15-18, W= 0.34, n.s. |
| | | male | 6-10 years | 21 | 2.38 | 0.79 | 2 | 0.673 | 0.01 | 6-10 vs. 11-14 , W= 1.15, n.s. |
| | | | 11-14 years | 58 | 2.57 | | | | | 6-10 vs 15-18, W= 1.19 n.s. |
| | | | 15-18 years | 51 | 2.55 | | | | | 11-14 vs 15-18, W= -0.05, n.s. |

| | response option | | age group | n | M | $\chi^2$ | df | p | ε² | DSCF |
|---|---|---|---|---|---|---|---|---|---|---|
| Do you currently have sleeping problems (during COVID-19)? | [1] yes<br>[2] no | | 6-10 years | 375 | 1.67 | | | | | 6-10 vs. 11-14 , W= -0.88, n.s. |
| | | all | 11-14 years | 952 | 1.67 | 27.73 | 2 | **< 0.001** | 0.01 | 6-10 > 15-18, W= -5.72, **p < 0.001** |
| | | | 15-18 years | 930 | 1.58 | | | | | 11-14 > 15-18, W= -6.51, **p < 0.001** |
| | | | 6-10 years | 187 | 1.63 | | | | | 6-10 vs. 11-14 , W= -0.92, n.s. |
| | | female | 11-14 years | 576 | 1.60 | 13.17 | 2 | **0.001** | 0.01 | 6-10 > 15-18, W= -3.91, **p = 0.016** |
| | | | 15-18 years | 676 | 1.51 | | | | | 11-14 > 15-18, W= -4.37, **p = 0.006** |
| | | | 6-10 years | 188 | 1.71 | | | | | 6-10 vs. 11-14 , W= 0.66, n.s. |
| | | male | 11-14 years | 376 | 1.73 | 6.00 | 2 | **0.050** | 0.01 | 6-10 vs. 15-18, W= -2.22, n.s. |
| | | | 15-18 years | 254 | 1.64 | | | | | 11-14 > 15-18, W= -3.39, **p = 0.044** |
| Type of sleeping problem (during COVID-19) | [1] I have problems falling asleep<br>[2] I have problems maintaining sleep<br>[3] I wake up at night, for example, and have problems falling asleep again<br>[4] I have nightmares and have difficulties falling asleep againg<br>[5] I wake up way before the alarm clock and have problems falling asleep again<br>[6] I have problems getting up when the alarm rings<br>[7] I sleep excessively<br>[8] I show unusual behavior, e.g., sleep walking, teeth grinding<br>[9] I show daytime sleepiness<br>[10] other | | 6-10 years | 124 | 3.16 | | | | | 6-10 vs. 11-14 , W= -0.99, n.s. |
| | | all | 11-14 years | 331 | 2.94 | 10.20 | 2 | **0.006** | 0.01 | 6-10 vs. 15-18, W= 2.19, n.s. |
| | | | 15-18 years | 421 | 3.26 | | | | | 11-14 < 15-18, W= 4.43, **p = 0.005** |
| | | | 6-10 years | 70 | 2.91 | | | | | 6-10 vs. 11-14 , W= 0.14, n.s. |
| | | female | 11-14 years | 230 | 3.06 | 10.89 | 2 | **0.004** | 0.02 | 6-10 vs. 15-18, W= 3.04, n.s. |
| | | | 15-18 years | 330 | 3.54 | | | | | 11-14 < 15-18, W= 4.22, **p = 0.008** |
| | | | 6-10 years | 54 | 3.41 | | | | | 6-10 vs. 11-14 , W= -1.74, n.s. |
| | | male | 11-14 years | 101 | 2.81 | 1.62 | 2 | 0.445 | 0.01 | 6-10 vs. 15-18, W= -1.21 n.s. |
| | | | 15-18 years | 91 | 2.97 | | | | | 11-14 vs. 15-18, W= 0.76, n.s. |
| How long have you had these sleeping problems? | [1] less than 1 month<br>[2] 1 month - 6 months<br>[3] 6 months - now | | 6-10 years | 124 | 2.36 | | | | | 6-10 vs. 11-14 , W= -0.26, n.s. |
| | | all | 11-14 years | 331 | 2.31 | 0.38 | 2 | 0.827 | 0.00 | 6-10 vs. 15-18, W= -0.79, n.s. |
| | | | 15-18 years | 421 | 2.32 | | | | | 11-14 vs. 15-18, W= -0.63, n.s. |
| | | | 6-10 years | 70 | 2.37 | | | | | 6-10 vs. 11-14 , W= -0.22, n.s. |
| | | female | 11-14 years | 230 | 2.33 | 0.88 | 2 | 0.644 | 0.00 | 6-10 vs. 15-18, W= -1.02, n.s. |
| | | | 15-18 years | 330 | 2.30 | | | | | 11-14 vs. 15-18, W= -1.10, n.s. |
| | | | 6-10 years | 54 | 2.35 | | | | | 6-10 vs. 11-14 , W= -0.32, n.s. |
| | | male | 11-14 years | 101 | 2.28 | 0.19 | 2 | 0.908 | 0.00 | 6-10 vs. 15-18, W= 0.26, n.s. |
| | | | 15-18 years | 91 | 2.34 | | | | | 11-14 vs. 15-18, W= 0.60, n.s. |
| How burdensome were these sleeping problems? | [1] not at all<br>[2] slightly<br>[3] moderately<br>[4] severely | | 6-10 years | 124 | 2.63 | | | | | 6-10 vs. 11-14 , W= 1.07, n.s. |
| | | all | 11-14 years | 331 | 2.65 | 5.62 | 2 | 0.060 | 0.01 | 6-10 vs. 15-18, W= 3.11, n.s. |
| | | | 15-18 years | 421 | 2.80 | | | | | 11-14 vs. 15-18, W= 2.36, n.s. |
| | | | 6-10 years | 70 | 2.69 | | | | | 6-10 vs. 11-14 , W= 1.06, n.s. |
| | | female | 11-14 years | 230 | 2.78 | 1.30 | 2 | 0.521 | 0.00 | 6-10 vs. 15-18, W= 1.65, n.s. |
| | | | 15-18 years | 330 | 2.82 | | | | | 11-14 vs. 15-18, W= 0.65, n.s. |
| | | | 6-10 years | 54 | 2.57 | | | | | 6-10 vs. 11-14 , W= -0.38, n.s. |
| | | male | 11-14 years | 101 | 2.51 | 5.01 | 2 | 0.082 | 0.02 | 6-10 vs. 15-18, W= 2.41, n.s. |
| | | | 15-18 years | 91 | 2.77 | | | | | 11-14 vs. 15-18, W= 2.90, n.s. |
| Does the current situation with COVID-19 scare you? | [1] yes, a lot<br>[2] yes, a bit<br>[3] hardly<br>[4] not at all | | 6-10 years | 375 | 2.63 | | | | | 6-10 < 11-14 , W= 4.79, **p = 0.002** |
| | | all | 11-14 years | 952 | 2.86 | 28.17 | 2 | **< 0.001** | 0.01 | 6-10 vs. 15-18, W= -0.20, n.s. |
| | | | 15-18 years | 930 | 2.67 | | | | | 11-14 > 15-18, W= -7.18, **p < 0.001** |
| | | | 6-10 years | 187 | 2.61 | | | | | 6-10 vs. 11-14 , W= 1.77, p = n.s. |
| | | female | 11-14 years | 576 | 2.71 | 9.49 | 2 | **0.009** | 0.01 | 6-10 vs. 15-18, W= -0.99, n.s. |
| | | | 15-18 years | 676 | 2.55 | | | | | 11-14 > 15-18, W= -4.39, **p = 0.005** |
| | | | 6-10 years | 188 | 2.64 | | | | | 6-10 < 11-14 , W= 5.84, **p < 0.001** |
| | | male | 11-14 years | 376 | 3.00 | 19.52 | 2 | **< 0.001** | 0.02 | 6-10 vs. 15-18, W= 2.14, n.s. |
| | | | 15-18 years | 254 | 2.78 | | | | | 11-14 > 15-18, W= -4.15, **p = 0.009** |

**Notes:** effect size ε²: < 0.04 very small to small effect; 0.04-0.16 medium effect; 0.16-0.64 large effect; > 0.64 very large effect; DSCF: Dwass-Steel-Critchlow-Fligner test corrects for multiple comparisons.

**Table S6.** Comparison between males and females for changes in type, duration and burden of sleeping problems before and during COVID-19 as well as COVID-19 related anxiety.

| | response option | age group | N | M | sex | n | M | U | p | r |
|---|---|---|---|---|---|---|---|---|---|---|
| How would you rate your general sleep quality during COVID-19 compared to before? | [1] a lot worse than before COVID-19<br>[2] worse than before COVID-19<br>[3] no change<br>[4] better than before COVID-19<br>[5] a lot better than before COVID-19 | 6-10 years | 375 | 2.69 | female | 187 | 2.64 | 16423.0 | 0.198 | 0.07 |
| | | | | | male | 188 | 2.73 | | | |
| | | 11-14 years | 952 | 2.70 | female | 576 | 2.63 | 99249.0 | **0.019** | 0.08 |
| | | | | | male | 376 | 2.76 | | | |
| | | 15-18 years | 930 | 2.63 | female | 676 | 2.57 | 79619.0 | 0.064 | 0.07 |
| | | | | | male | 254 | 2.69 | | | |
| | | 6-18 years | 2257 | 2.67 | female | 1439 | 2.60 | 539415.0 | **< 0.001** | 0.08 |
| | | | | | male | 818 | 2.73 | | | |
| Did you have sleeping problems in the past (before COVID-19)? | [1] yes<br>[2] no | 6-10 years | 375 | 1.87 | female | 187 | 1.84 | 16816.0 | 0.218 | 0.04 |
| | | | | | male | 188 | 1.89 | | | |
| | | 11-14 years | 952 | 1.81 | female | 576 | 1.77 | 99612.0 | **0.003** | 0.08 |
| | | | | | male | 376 | 1.85 | | | |
| | | 15-18 years | 930 | 1.75 | female | 676 | 1.69 | 76293.0 | **< 0.001** | 0.11 |
| | | | | | male | 254 | 1.80 | | | |
| | | 6-18 years | 2257 | 1.79 | female | 1439 | 1.74 | 528711.0 | **< 0.001** | 0.10 |
| | | | | | male | 818 | 1.84 | | | |
| Type of sleeping problem (before COVID-19)? | [1] I had problems falling asleep<br>[2] I had problems maintaining sleep<br>[3] I woke up at night, for example, and had problems falling asleep again<br>[4] I had nightmares and had difficulties falling asleep again<br>[5] I woke up way before the alarm clock and had problems falling asleep again<br>[6] I had problems getting up when the alarm rang<br>[7] I slept excessively<br>[8] I showed unusual behavior, e.g., sleep walking, teeth grinding<br>[9] I showed daytime sleepiness<br>[10] other | 6-10 years | 50 | 2.45 | female | 29 | 2.52 | 250.5 | 0.253 | 0.18 |
| | | | | | male | 21 | 2.38 | | | |
| | | 11-14 years | 193 | 2.34 | female | 135 | 2.81 | 3269.0 | **0.045** | 0.17 |
| | | | | | male | 58 | 1.86 | | | |
| | | 15-18 years | 262 | 2.61 | female | 211 | 2.61 | 5273.0 | 0.811 | 0.02 |
| | | | | | male | 51 | 2.61 | | | |
| | | 6-18 years | 505 | 2.46 | female | 375 | 2.67 | 21729.0 | **0.045** | 0.11 |
| | | | | | male | 130 | 2.24 | | | |
| How long have you had these sleeping problems? | [1] less than 1 month<br>[2] 1 month - 6 months<br>[3] 6 months - 2 years<br>[4] 2 years - 5 years<br>[5] more than 5 years | 6-10 years | 50 | 2.98 | female | 29 | 2.86 | 271.0 | 0.502 | 0.11 |
| | | | | | male | 21 | 3.10 | | | |
| | | 11-14 years | 193 | 3.04 | female | 135 | 3.04 | 3891.0 | 0.946 | 0.01 |
| | | | | | male | 58 | 3.03 | | | |
| | | 15-18 years | 262 | 3.07 | female | 211 | 2.96 | 4718.0 | 0.156 | 0.12 |
| | | | | | male | 51 | 3.18 | | | |
| | | 6-18 years | 505 | 3.04 | female | 375 | 2.98 | 22951.0 | 0.305 | 0.06 |
| | | | | | male | 130 | 3.10 | | | |
| How burdensome were these sleeping problems? | [1] not at all<br>[2] slightly<br>[3] moderately<br>[4] severely | 6-10 years | 50 | 2.50 | female | 29 | 2.62 | 268.5 | 0.454 | 0.12 |
| | | | | | male | 21 | 2.38 | | | |
| | | 11-14 years | 193 | 2.64 | female | 135 | 2.71 | 3571.0 | 0.312 | 0.09 |
| | | | | | male | 58 | 2.57 | | | |
| | | 15-18 years | 262 | 2.65 | female | 211 | 2.74 | 4671.0 | 0.116 | 0.13 |
| | | | | | male | 51 | 2.55 | | | |
| | | 6-18 years | 505 | 2.63 | female | 375 | 2.72 | 21313.0 | **0.024** | 0.13 |
| | | | | | male | 130 | 2.53 | | | |

| | response option | age group | N | M | sex | n | M | U | p | r |
|---|---|---|---|---|---|---|---|---|---|---|
| Do you currently have sleeping problems (during COVID-19)? | [1] yes<br>[2] no | 6-10 years | 375 | 1.67 | female | 187 | 1.63 | 16047.0 | 0.074 | 0.09 |
| | | | | | male | 188 | 1.71 | | | |
| | | 11-14 years | 952 | 1.67 | female | 576 | 1.60 | 93948.0 | **< 0.001** | 0.13 |
| | | | | | male | 376 | 1.73 | | | |
| | | 15-18 years | 930 | 1.58 | female | 676 | 1.51 | 74700.0 | **< 0.001** | 0.13 |
| | | | | | male | 254 | 1.64 | | | |
| | | 6-18 years | 2257 | 1.63 | female | 1439 | 1.56 | 507469.0 | **< 0.001** | 0.14 |
| | | | | | male | 818 | 1.70 | | | |
| Type of sleeping problem (during COVID-19) | [1] I have problems falling asleep<br>[2] I have problems maintaining sleep<br>[3] I wake up at night, for example, and have problems falling asleep again<br>[4] I have nightmares and have difficulties falling asleep againg<br>[5] I wake up way before the alarm clock and have problems falling asleep again<br>[6] I have problems getting up when the alarm rings<br>[7] I sleep excessively<br>[8] I show unusual behavior, e.g., sleep walking, teeth grinding<br>[9] I show daytime sleepiness<br>[10] other | 6-10 years | 124 | 3.16 | female | 70 | 2.91 | 1750.5 | 0.466 | 0.07 |
| | | | | | male | 54 | 3.41 | | | |
| | | 11-14 years | 331 | 2.94 | female | 230 | 3.06 | 11039.0 | 0.413 | 0.05 |
| | | | | | male | 101 | 2.81 | | | |
| | | 15-18 years | 421 | 3.26 | female | 330 | 3.54 | 12821.0 | **0.029** | 0.15 |
| | | | | | male | 91 | 2.97 | | | |
| | | 6-18 years | 877 | 3.15 | female | 631 | 3.29 | 71051.0 | **0.044** | 0.08 |
| | | | | | male | 246 | 3.00 | | | |
| How long have you had these sleeping problems? | [1] less than 1 month<br>[2] 1 month - 6 months<br>[3] 6 months - now | 6-10 years | 124 | 2.36 | female | 70 | 2.37 | 1835.0 | 0.759 | 0.03 |
| | | | | | male | 54 | 2.35 | | | |
| | | 11-14 years | 331 | 2.31 | female | 230 | 2.33 | 11253.0 | 0.623 | 0.03 |
| | | | | | male | 101 | 2.28 | | | |
| | | 15-18 years | 421 | 2.32 | female | 330 | 2.30 | 14462.0 | 0.555 | 0.04 |
| | | | | | male | 91 | 2.34 | | | |
| | | 6-18 years | 877 | 2.32 | female | 631 | 2.32 | 77402.0 | 0.977 | 0.00 |
| | | | | | male | 246 | 2.32 | | | |
| How burdensome were these sleeping problems? | [1] not at all<br>[2] slightly<br>[3] moderately<br>[4] severely | 6-10 years | 124 | 2.63 | female | 70 | 2.69 | 1727.5 | 0.383 | 0.09 |
| | | | | | male | 54 | 2.57 | | | |
| | | 11-14 years | 331 | 2.65 | female | 230 | 2.78 | 9876.0 | **0.019** | 0.15 |
| | | | | | male | 101 | 2.51 | | | |
| | | 15-18 years | 421 | 2.80 | female | 330 | 2.82 | 14704.0 | 0.744 | 0.02 |
| | | | | | male | 91 | 2.77 | | | |
| | | 6-18 years | 877 | 2.71 | female | 631 | 2.79 | 69572.0 | **0.011** | 0.09 |
| | | | | | male | 246 | 2.62 | | | |
| Does the current situation with COVID-19 scare you? | [1] yes, a lot<br>[2] yes, a bit<br>[3] hardly<br>[4] not at all | 6-10 years | 375 | 2.63 | female | 187 | 2.61 | 17283.0 | 0.769 | 0.02 |
| | | | | | male | 188 | 2.64 | | | |
| | | 11-14 years | 952 | 2.86 | female | 576 | 2.71 | 88972.0 | **< 0.001** | 0.18 |
| | | | | | male | 376 | 3.00 | | | |
| | | 15-18 years | 930 | 2.67 | female | 676 | 2.55 | 73279.0 | **< 0.001** | 0.15 |
| | | | | | male | 254 | 2.78 | | | |
| | | 6-18 years | 2257 | 2.74 | female | 1439 | 2.62 | 505831.0 | **< 0.001** | 0.14 |
| | | | | | male | 818 | 2.85 | | | |

**Notes:** effect size r = biserial rank correlation: < 0.28 very small to small effect; 0.28 – 0.43 medium effect; > 0.43 large effect

**Table S7.** COVID-19 related anxiety as well as general sleep quality and sleeping problems within the response categories of COVID-19 related anxiety (yes, a lot; yes, a bit; hardly; not at all).

| | | 6-10 years | | | | | 11-14 years | | | | | 15-18 years | | | | |
|---|---|---|---|---|---|---|---|---|---|---|---|---|---|---|---|---|
| | | | | | 95% CI | | | | | 95% CI | | | | | 95% CI | |
| | **response option** | **n** | **N** | **%** | **Lower** | **Upper** | **n** | **N** | **%** | **Lower** | **Upper** | **n** | **N** | **%** | **Lower** | **Upper** |
| Does the current situation with COVID-19 scare you? | [1] yes, a lot | 48 | 375 | 12.8 | 9.59 | 16.61 | 73 | 952 | 7.7 | 6.06 | 9.55 | 98 | 930 | 10.5 | 8.64 | 12.69 |
| | [2] yes, a bit | 137 | 375 | 36.5 | 31.65 | 41.63 | 271 | 952 | 28.5 | 25.62 | 31.45 | 339 | 930 | 36.5 | 33.35 | 39.64 |
| | [3] hardly | 96 | 375 | 25.6 | 21.26 | 30.33 | 357 | 952 | 37.5 | 34.42 | 40.66 | 316 | 930 | 34.0 | 30.94 | 37.12 |
| | [4] not at all | 94 | 375 | 25.1 | 20.76 | 29.77 | 251 | 952 | 26.4 | 23.59 | 29.29 | 177 | 930 | 19.0 | 16.56 | 21.71 |
| General sleep quality | [1] a lot worse than before COVID-19; [1] yes a lot | 9 | 48 | 18.8 | 8.95 | 32.60 | 15 | 73 | 20.5 | 11.98 | 31.62 | 23 | 98 | 23.5 | 15.50 | 33.10 |
| | [1] a lot worse than before COVID-19; [1] yes, a bit | 5 | 137 | 3.6 | 1.20 | 8.31 | 37 | 271 | 13.7 | 9.80 | 18.32 | 34 | 339 | 10.0 | 7.05 | 13.73 |
| | [1] a lot worse than before COVID-19; [3] hardly | 1 | 96 | 1.0 | 0.03 | 5.67 | 13 | 357 | 3.6 | 1.95 | 6.15 | 16 | 316 | 5.1 | 2.92 | 8.09 |
| | [1] a lot worse than before COVID-19; [4] not at all | | | | | | 19 | 250 | 7.6 | 4.64 | 11.61 | 19 | 177 | 10.7 | 6.59 | 16.25 |
| | [2] worse than before COVID-19; [1] yes, a lot | 23 | 48 | 47.9 | 33.29 | 62.80 | 30 | 73 | 41.1 | 29.71 | 53.23 | 40 | 98 | 40.8 | 30.99 | 51.20 |
| | [2] worse than before COVID-19; [2] yes, a bit | 51 | 137 | 37.2 | 29.13 | 45.89 | 93 | 271 | 34.3 | 28.68 | 40.30 | 115 | 339 | 33.9 | 28.90 | 39.23 |
| | [2] worse than before COVID-19; [3] hardly | 21 | 96 | 21.9 | 14.08 | 31.47 | 78 | 357 | 21.8 | 17.67 | 26.50 | 103 | 316 | 32.6 | 27.45 | 38.07 |
| | [2] worse than before COVID-19; [4] not at all | 12 | 94 | 12.8 | 6.77 | 21.20 | 48 | 250 | 19.2 | 14.51 | 24.64 | 32 | 177 | 18.1 | 12.71 | 24.55 |
| | [3] no change;[1] yes, a lot | 14 | 48 | 29.2 | 16.95 | 44.10 | 25 | 73 | 34.2 | 23.53 | 46.28 | 31 | 98 | 31.6 | 22.61 | 41.80 |
| | [3] no change;[2] yes, a bit | 77 | 137 | 56.2 | 47.48 | 64.66 | 121 | 271 | 44.6 | 38.63 | 50.78 | 151 | 339 | 44.5 | 39.17 | 50.01 |
| | [3] no change; [3] hardly | 69 | 96 | 71.9 | 61.78 | 80.58 | 230 | 357 | 64.4 | 59.22 | 69.39 | 169 | 316 | 53.5 | 47.81 | 59.08 |
| | [3] no change;[4] not at all | 75 | 94 | 79.8 | 70.25 | 87.40 | 152 | 250 | 60.8 | 54.45 | 66.89 | 107 | 177 | 60.5 | 52.84 | 67.71 |
| | [4] better than before COVID-19; [1] yes, a lot | 2 | 48 | 4.2 | 0.51 | 14.30 | 2 | 73 | 2.7 | 0.33 | 9.55 | 4 | 98 | 4.1 | 1.12 | 10.10 |
| | [4] better than before COVID-19; [2] yes, a bit | 3 | 137 | 2.2 | 0.45 | 6.27 | 18 | 271 | 6.6 | 3.98 | 10.29 | 32 | 339 | 9.4 | 6.55 | 13.06 |
| | [4] better than before COVID-19; [3] hardly | 5 | 96 | 5.2 | 1.71 | 11.74 | 31 | 357 | 8.7 | 5.98 | 12.10 | 25 | 316 | 7.9 | 5.19 | 11.46 |
| | [4] better than before COVID-19; [4] not at all | 7 | 94 | 7.4 | 3.05 | 14.70 | 17 | 250 | 6.8 | 4.01 | 10.66 | 14 | 177 | 7.9 | 4.39 | 12.91 |
| | [5] a lot better than before COVID-19; [1] yes, a lot | | | 0.0 | 0.00 | 0.00 | 1 | 73 | 1.4 | 0.03 | 7.40 | | | | | |
| | [5] a lot better than before COVID-19; [2] yes, a bit | 1 | 137 | 0.7 | 0.02 | 4.00 | 2 | 271 | 0.7 | 0.09 | 2.64 | 7 | 339 | 2.1 | 0.83 | 4.21 |
| | [5] a lot better than before COVID-19; [3] hardly | | | | | | 5 | 357 | 1.4 | 0.46 | 3.24 | 3 | 316 | 0.9 | 0.20 | 2.75 |
| | [5] a lot better than before COVID-19; [4] not at all | | | | | | 14 | 250 | 5.6 | 3.10 | 9.22 | 5 | 177 | 2.8 | 0.92 | 6.47 |
| Sleeping problems before COVID-19 per COVID-19 anxiety category | [1] yes; [1] yes, a lot | 6 | 48 | 12.5 | 4.73 | 25.20 | 15 | 73 | 20.5 | 12.00 | 31.60 | 29 | 98 | 29.6 | 20.80 | 39.70 |
| | [1] yes; [2] yes, a bit | 17 | 137 | 12.4 | 7.40 | 19.10 | 63 | 271 | 23.2 | 18.40 | 28.70 | 102 | 339 | 30.1 | 25.30 | 35.30 |
| | [1] yes; [3] hardly | 15 | 96 | 15.6 | 9.02 | 24.50 | 64 | 357 | 17.9 | 14.10 | 22.30 | 82 | 316 | 25.9 | 21.20 | 31.20 |
| | [1] yes; [4] not at all | 12 | 94 | 12.8 | 6.77 | 21.20 | 51 | 251 | 20.3 | 15.50 | 25.80 | 49 | 177 | 27.7 | 21.20 | 34.90 |
| | [2] no; [1] yes, a lot | 42 | 48 | 87.5 | 74.75 | 95.30 | 58 | 73 | 79.5 | 68.40 | 88.00 | 69 | 98 | 70.4 | 60.30 | 79.20 |
| | [2] no; [2] yes, a bit | 120 | 137 | 87.6 | 80.88 | 92.60 | 208 | 271 | 76.8 | 71.30 | 81.60 | 237 | 339 | 69.9 | 64.70 | 74.70 |
| | [2] no; [3] hardly | 81 | 96 | 84.4 | 75.54 | 91.00 | 293 | 357 | 82.1 | 77.70 | 85.90 | 234 | 316 | 74.1 | 68.80 | 78.80 |
| | [2] no; [4] not at all | 82 | 94 | 87.2 | 78.76 | 93.20 | 200 | 251 | 79.7 | 74.20 | 84.50 | 128 | 177 | 72.3 | 65.10 | 78.80 |

| | | 6-10 years | | | | | 11-14 years | | | | | 15-18 years | | | | |
|---|---|---|---|---|---|---|---|---|---|---|---|---|---|---|---|---|
| | | | | | 95% CI | | | | | 95% CI | | | | | 95% CI | |
| | response option | n | N | % | Lower | Upper | n | N | % | Lower | Upper | n | N | % | Lower | Upper |
| Sleeping problems during COVID-19 per COVID-19 anxiety category | [1] yes; [1] yes, a lot | 30 | 48 | 62.5 | 47.35 | 76.00 | 47 | 73 | 64.4 | 52.30 | 75.30 | 67 | 98 | 68.4 | 58.20 | 77.40 |
| | [1] yes; [2] yes, a bit | 57 | 137 | 41.6 | 33.25 | 50.30 | 124 | 271 | 45.8 | 39.70 | 51.90 | 162 | 339 | 47.8 | 42.40 | 53.30 |
| | [1] yes; [3] hardly | 25 | 96 | 26.0 | 17.62 | 36.00 | 92 | 357 | 25.8 | 21.30 | 30.60 | 128 | 316 | 40.5 | 35.00 | 46.10 |
| | [1] yes; [4] not at all | 12 | 94 | 12.8 | 6.77 | 21.20 | 69 | 251 | 27.5 | 22.10 | 33.50 | 64 | 177 | 36.2 | 29.10 | 43.70 |
| | [1] no; [1] yes, a lot | 18 | 48 | 37.5 | 23.95 | 52.60 | 26 | 73 | 35.6 | 24.70 | 47.70 | 31 | 98 | 31.6 | 22.60 | 41.80 |
| | [2] no; [2] yes, a bit | 80 | 137 | 58.4 | 49.67 | 66.70 | 147 | 271 | 54.2 | 48.10 | 60.30 | 177 | 339 | 52.2 | 46.70 | 57.60 |
| | [2] no; [3] hardly | 71 | 96 | 74.0 | 64.00 | 82.40 | 265 | 357 | 74.2 | 69.40 | 78.70 | 188 | 316 | 59.5 | 53.90 | 65.00 |
| | [2] no; [4] not at all | 82 | 94 | 87.2 | 78.76 | 93.20 | 182 | 251 | 72.5 | 66.50 | 77.90 | 113 | 177 | 63.8 | 56.30 | 70.90 |

**Notes:** CI, 95% confidence interval with lower and upper border; empty spaces: response option was not chosen in this age group

**Table S8**. COVID-19 related anxiety as well as general sleep quality and sleeping problems in females and males within the response categories of COVID-19 related anxiety (yes, a lot; yes, a bit; hardly; not at all).

| | | | | How would you rate your general sleep quality during COVID-19 compared to before? | | | | | Did you have sleeping problems in the past (before COVID-19)? [1] Yes, [2] No | | | | | Do you currently have sleeping problems (during COVID-19)? [1] Yes, [2] No | | | | | Sleeping problems before COVID-19 vs. during COVID-19 | | | |
|---|---|---|---|---|---|---|---|---|---|---|---|---|---|---|---|---|---|---|---|---|---|---|
| | | | | **n** | **M** | **U** | **p** | **r** | **n** | **M** | **U** | **p** | **r** | **n** | **M** | **U** | **p** | **r** | **n** | **w** | **p** | **r** |
| General sleep quality and sleeping problems | 6-10 years | | all | 375 | 2.69 | | | | 375 | 1.87 | | | | 375 | 1.67 | | | | 375 | 4673 | **< 0.001** | 0.71 |
| | | | female | 187 | 2.64 | 16423 | 0.198 | 0.07 | 187 | 1.84 | 16816 | 0.218 | 0.04 | 187 | 1.63 | 16047 | 0.074 | 0.09 | 187 | 1344 | **< 0.001** | 0.75 |
| | | | male | 188 | 2.73 | | | | 188 | 1.89 | | | | 188 | 1.71 | | | | 188 | 1025 | **< 0.001** | 0.67 |
| | 11-14 years | | all | 951 | 2.68 | | | | 951 | 1.81 | | | | 951 | 1.67 | | | | 952 | 32850 | **< 0.001** | 0.47 |
| | | | female | 576 | 2.63 | 99249 | **0.019** | 0.08 | 576 | 1.77 | 99612 | **0.003** | 0.08 | 576 | 1.60 | 93948 | **< 0.001** | 0.13 | 576 | 15124 | **< 0.001** | 0.48 |
| | | | male | 376 | 2.76 | | | | 376 | 1.85 | | | | 376 | 1.73 | | | | 376 | 3430 | **< 0.001** | 0.44 |
| | 15-18 years | | all | 930 | 2.60 | | | | 930 | 1.75 | | | | 930 | 1.58 | | | | 730 | 36270 | **< 0.001** | 0.52 |
| | | | female | 676 | 2.57 | 79619 | 0.064 | 0.07 | 676 | 1.69 | 76293 | **< 0.001** | 0.11 | 676 | 1.51 | 74700 | **< 0.001** | 0.13 | 676 | 21480 | **< 0.001** | 0.50 |
| | | | male | 254 | 2.69 | | | | 54 | 1.80 | | | | 54 | 1.64 | | | | 54 | 1952.5 | **< 0.001** | 0.57 |
| | 6-18 years | | all | 2257 | 2.67 | | | | 2257 | 1.79 | | | | 2257 | 1.63 | | | | 2257 | 193223 | **< 0.001** | 0.52 |
| | | | female | 1439 | 2.60 | 539415 | **< 0.001** | 0.08 | 1439 | 1.74 | 528711 | **< 0.001** | 0.10 | 1439 | 1.56 | 507469 | **< 0.001** | 0.14 | 1439 | 93436 | **< 0.001** | 0.52 |
| | | | male | 818 | 2.73 | | | | 818 | 1.84 | | | | 818 | 1.70 | | | | 818 | 18011 | **< 0.001** | 0.54 |
| | | | | **n** | **M** | **U** | **p** | **r** | **n** | **M** | **U** | **p** | **r** | **n** | **M** | **U** | **p** | **r** | **n** | **w** | **p** | **r** |
| Does the current situation with COVID-19 scare you? | 6-10 years | [1] yes, a lot | all | 48 | 2.19 | | | | 48 | 1.88 | | | | 48 | 1.38 | | | | 48 | 377 | **< 0.001** | 0.86 |
| | | | female | 23 | 2.13 | 265 | 0.616 | 0.08 | 23 | 1.83 | 261 | 0.340 | 0.09 | 23 | 1.35 | 273 | 0.721 | 0.05 | 23 | 84 | **0.003** | 0.85 |
| | | | male | 25 | 2.24 | | | | 25 | 1.92 | | | | 25 | 1.40 | | | | 25 | 112 | **< 0.001** | 0.87 |
| | | [2] yes, a bit | all | 137 | 2.60 | | | | 137 | 1.88 | | | | 137 | 1.59 | | | | 137 | 945 | **< 0.001** | 0.91 |
| | | | female | 71 | 2.55 | 2175 | 0.411 | 0.07 | 71 | 1.85 | 2193 | 0.259 | 0.06 | 71 | 1.54 | 2106 | 0.233 | 0.10 | 71 | 324 | **< 0.001** | 0.85 |
| | | | male | 66 | 2.64 | | | | 66 | 1.91 | | | | 66 | 1.64 | | | | 66 | 171 | **< 0.001** | 1.00 |
| | | [3] hardly | all | 96 | 2.81 | | | | 96 | 1.85 | | | | 96 | 1.74 | | | | 96 | 133 | **0.020** | 0.56 |
| | | | female | 48 | 2.77 | 1083 | 0.523 | 0.06 | 48 | 1.81 | 1080 | 0.405 | 0.06 | 48 | 1.71 | 1080 | 0.491 | 0.06 | 48 | 48 | 0.145 | 0.46 |
| | | | male | 48 | 2.85 | | | | 48 | 1.88 | | | | 48 | 1.77 | | | | 48 | 24 | 0.073 | 0.71 |
| | | [4] not at all | all | 94 | 2.95 | | | | 94 | 1.88 | | | | 94 | 1.87 | | | | 94 | 52.5 | 1.000 | 0.00 |
| | | | female | 45 | 2.91 | 1034 | 0.459 | 0.06 | 45 | 1.89 | 1068 | 0.651 | 0.03 | 45 | 1.82 | 997 | 0.167 | 0.10 | 45 | 12 | 0.233 | 0.60 |
| | | | male | 49 | 2.98 | | | | 49 | 1.86 | | | | 49 | 1.92 | | | | 49 | 15 | 0.351 | 0.33 |
| | 11-14 years | [1] yes, a lot | all | 73 | 2.20 | | | | 73 | 1.78 | | | | 73 | 1.35 | | | | 73 | 796 | **< 0.001** | 0.76 |
| | | | female | 54 | 2.28 | 451 | 0.407 | 0.12 | 54 | 1.81 | 473 | 0.478 | 0.08 | 54 | 1.37 | 485 | 0.677 | 0.05 | 54 | 462 | **< 0.001** | 0.75 |
| | | | male | 19 | 2.11 | | | | 19 | 1.74 | | | | 19 | 1.32 | | | | 19 | 49.5 | **0.013** | 0.80 |
| | | [2] yes, a bit | all | 271 | 2.53 | | | | 271 | 1.79 | | | | 271 | 1.56 | | | | 271 | 3744 | **< 0.001** | 0.64 |
| | | | female | 192 | 2.39 | 6454 | **0.039** | 0.15 | 192 | 1.73 | 6722 | **0.045** | 0.11 | 192 | 1.52 | 7022 | 0.267 | 0.07 | 192 | 1836 | **< 0.001** | 0.61 |
| | | | male | 79 | 2.66 | | | | 79 | 1.85 | | | | 79 | 1.59 | | | | 79 | 348 | **< 0.001** | 0.71 |
| | | [3] hardly | all | 357 | 2.82 | | | | 357 | 1.83 | | | | 357 | 1.75 | | | | 357 | 3007 | **0.004** | 0.29 |
| | | | female | 197 | 2.83 | 15759 | 0.999 | 0.00 | 197 | 1.78 | 14389 | **0.033** | 0.09 | 197 | 1.68 | 13577 | **0.003** | 0.14 | 197 | 1518 | **0.015** | 0.29 |
| | | | male | 160 | 2.81 | | | | 160 | 1.87 | | | | 160 | 1.82 | | | | 160 | 261 | 0.134 | 0.29 |
| | | [4] not at all | all | 250 | 2.84 | | | | 251 | 1.80 | | | | 251 | 1.73 | | | | 251 | 1407 | **0.027** | 0.27 |
| | | | female | 133 | 2.80 | 7618 | 0.745 | 0.02 | 133 | 1.77 | 7348 | 0.213 | 0.06 | 133 | 1.68 | 7165 | 0.124 | 0.09 | 133 | 414 | 0.064 | 0.31 |
| | | | male | 117 | 2.87 | | | | 118 | 1.83 | | | | 118 | 1.77 | | | | 118 | 304 | 0.213 | 0.23 |

| | | | | How would you rate your general sleep quality during COVID-19 compared to before? | | | | | Did you have sleeping problems in the past (before COVID-19)? [1] Yes, [2] No | | | | | Do you currently have sleeping problems (during COVID-19)? [1] Yes, [2] No | | | | | Sleeping problems before COVID-19 vs. during COVID-19 | | | |
|---|---|---|---|---|---|---|---|---|---|---|---|---|---|---|---|---|---|---|---|---|---|---|
| | | | | **n** | **M** | **U** | **p** | **r** | **n** | **M** | **U** | **p** | **r** | **n** | **M** | **U** | **p** | **r** | **n** | **w** | **p** | **r** |
| Does the current situation with COVID-19 scare you? | 15-18 years | [1] yes, a lot | all | 98 | 2.23 | | | | 98 | 1.76 | | | | 98 | 1.36 | | | | 98 | 987 | **< 0.001** | 0.83 |
| | | | female | 72 | 2.08 | 735 | 0.087 | 0.22 | 72 | 1.64 | 706 | **0.020** | 0.25 | 72 | 1.26 | 751 | 0.065 | 0.20 | 72 | 510 | **< 0.001** | 0.82 |
| | | | male | 26 | 2.38 | | | | 26 | 1.88 | | | | 26 | 1.46 | | | | 26 | 84 | **0.003** | 0.85 |
| | | [2] yes, a bit | all | 339 | 2.64 | | | | 339 | 1.74 | | | | 339 | 1.74 | | | | 339 | 6063 | **< 0.001** | 0.47 |
| | | | female | 274 | 2.57 | 7990 | 0.160 | 0.10 | 274 | 1.68 | 7794 | **0.049** | 0.13 | 274 | 1.68 | 7708 | 0.052 | 0.13 | 274 | 4212 | **< 0.001** | 0.46 |
| | | | male | 65 | 2.71 | | | | 65 | 1.80 | | | | 65 | 1.80 | | | | 65 | 176 | **0.017** | 0.52 |
| | | [3] hardly | all | 316 | 2.67 | | | | 316 | 1.77 | | | | 316 | 1.62 | | | | 316 | 2982 | **< 0.001** | 0.52 |
| | | | female | 214 | 2.69 | 10442 | 0.491 | 0.04 | 214 | 1.69 | 9260 | **0.004** | 0.15 | 214 | 1.55 | 9442 | **0.023** | 0.14 | 214 | 1528 | **< 0.001** | 0.47 |
| | | | male | 102 | 2.64 | | | | 102 | 1.84 | | | | 102 | 1.69 | | | | 102 | 250 | **0.001** | 0.67 |
| | | [4] not at all | all | 177 | 2.77 | | | | 177 | 1.72 | | | | 177 | 1.65 | | | | 177 | 744 | **0.029** | 0.32 |
| | | | female | 116 | 2.67 | 3238 | 0.293 | 0.08 | 116 | 1.74 | 3351 | 0.458 | 0.05 | 116 | 1.63 | 3445 | 0.730 | 0.03 | 116 | 432 | **0.029** | 0.37 |
| | | | male | 61 | 2.87 | | | | 61 | 1.69 | | | | 61 | 1.66 | | | | 61 | 45.5 | 0.594 | 0.17 |
| | 6-18 years | [1] yes, a lot | all | 219 | 2.21 | | | | 219 | 1.80 | | | | 219 | 1.36 | | | | 219 | 6143 | **< 0.001** | 0.81 |
| | | | female | 149 | 2.16 | 4846 | 0.369 | 0.07 | 149 | 1.73 | 4560 | **0.040** | 0.13 | 149 | 1.32 | 4774 | 0.220 | 0.08 | 149 | 2765 | **< 0.001** | 0.79 |
| | | | male | 70 | 2.26 | | | | 70 | 1.86 | | | | 70 | 1.40 | | | | 70 | 683 | **< 0.001** | 0.84 |
| | | [2] yes, a bit | all | 747 | 2.59 | | | | 747 | 1.79 | | | | 747 | 1.57 | | | | 747 | 28676 | **< 0.001** | 0.60 |
| | | | female | 537 | 2.50 | 50378 | **0.014** | 0.11 | 537 | 1.72 | 48854 | **< 0.001** | 0.13 | 537 | 1.51 | 50250 | **0.007** | 0.11 | 537 | 15678 | **< 0.001** | 0.56 |
| | | | male | 210 | 2.67 | | | | 210 | 1.85 | | | | 210 | 1.62 | | | | 210 | 1972 | **< 0.001** | 0.73 |
| | | [3] hardly | all | 769 | 2.76 | | | | 769 | 1.80 | | | | 769 | 1.70 | | | | 769 | 14515 | **< 0.001** | 0.42 |
| | | | female | 459 | 2.76 | 70636 | 0.847 | 0.01 | 459 | 1.74 | 62724 | **< 0.001** | 0.12 | 459 | 1.62 | 60854 | **< 0.001** | 0.15 | 459 | 7125 | **< 0.001** | 0.39 |
| | | | male | 310 | 2.76 | | | | 310 | 1.86 | | | | 310 | 1.77 | | | | 310 | 1320 | **< 0.001** | 0.49 |
| | | [4] not at all | all | 521 | 2.83 | | | | 522 | 1.79 | | | | 522 | 1.73 | | | | 522 | 5120 | **0.003** | 0.26 |
| | | | female | 294 | 2.77 | 31474 | 0.193 | 0.06 | 294 | 1.78 | 32754 | 0.531 | 0.23 | 294 | 1.68 | 30558 | **0.026** | 0.09 | 294 | 1938 | **0.002** | 0.36 |
| | | | male | 227 | 2.89 | | | | 228 | 1.80 | | | | 228 | 1.77 | | | | 228 | 768.5 | 0.480 | 0.12 |

**Notes:** effect size r = biserial rank correlation: < 0.28 very small to small effect; 0.28 – 0.43 medium effect; > 0.43 large effect

**Table S9**. Comparison between age groups for changes in time spent in bed, regularity of bedtimes and daytime sleep/napping.

| | response option | | age group | n | M | $\chi^2$ | df | p | ε² | DSCF |
|---|---|---|---|---|---|---|---|---|---|---|
| How did the time you usually spend in bed change? | [1] a lot shorter<br>[2] considerably shorter<br>[3] sligthly shorter<br>[4] no change<br>[5] slightly longer<br>[6] considerably longer<br>[7] a lot longer | all | 6-10 years | 375 | 4.19 | 100.15 | 2 | **< 0.001** | 0.04 | 6-10 < 11-14 , W= 5.86, **p < 0.001** |
| | | | 11-14 years | 952 | 4.43 | | | | | 6-10 < 15-18, W= 13.48, **p < 0.001** |
| | | | 15-18 years | 930 | 4.72 | | | | | 11-14 < 15-18, W= 9.49, **p < 0.001** |
| | | female | 6-10 years | 187 | 4.22 | 72.83 | 2 | **< 0.001** | 0.05 | 6-10 < 11-14 , W=3.99, **p = 0.013** |
| | | | 11-14 years | 576 | 4.46 | | | | | 6-10 < 15-18, W= 11.05, **p < 0.001** |
| | | | 15-18 years | 676 | 4.94 | | | | | 11-14 < 15-18, W= 8.61, **p < 0.001** |
| | | male | 6-10 years | 188 | 4.15 | 15.11 | 2 | **< 0.001** | 0.02 | 6-10 < 11-14 , W= 4:18, **p = 0.009** |
| | | | 11-14 years | 376 | 4.39 | | | | | 6-10 < 15-18, W= 5.14, **p < 0.001** |
| | | | 15-18 years | 254 | 4.49 | | | | | 11-14 vs. 15-18, W= 2.34, n.s. |
| Do you/did you go to bed at the same time every day (+/-30min)? This question refers to the time during COVID-19 as well as to the normal state before COVID-19 | [1] yes<br>[2] no | all | 6-10 years | 375 | 1.15 | 109.55 | 2 | **< 0.001** | 0.05 | 6-10 < 11-14 , W= 9.85, **p < 0.001** |
| | | | 11-14 years | 952 | 1.34 | | | | | 6-10 < 15-18, W= 14.60, **p < 0.001** |
| | | | 15-18 years | 930 | 1.46 | | | | | 11-14 < 15-18, W= 7.05, **p < 0.001** |
| | | female | 6-10 years | 187 | 1.14 | 57.33 | 2 | **< 0.001** | 0.04 | 6-10 < 11-14 , W= 7.93, **p < 0.001** |
| | | | 11-14 years | 576 | 1.36 | | | | | 6-10 < 15-18, W= 10.62, **p < 0.001** |
| | | | 15-18 years | 676 | 1.44 | | | | | 11-14 < 15-18, W= 4.42, **p = 0.008** |
| | | male | 6-10 years | 188 | 1.15 | 52.93 | 2 | **< 0.001** | 0.06 | 6-10 < 11-14 , W= 5.67, **p < 0.001** |
| | | | 11-14 years | 376 | 1.31 | | | | | 6-10 < 15-18, W= 10.09, **p < 0.001** |
| | | | 15-18 years | 254 | 1.48 | | | | | 11-14 < 15-18, W= 6.06, **p < 0.001** |
| | [1] my bedtime is irregular independently of COVID-19<br>[2] my bedtime was regular before and got irregular during COVID-19<br>[3] my bedtime was irregular before and got regular during COVID-19 | all | 6-10 years | 55 | 1.49 | 14.06 | 2 | **< 0.001** | 0.02 | 6-10 < 11-14 , W= 3.44, **p = 0.040** |
| | | | 11-14 years | 322 | 1.79 | | | | | 6-10 < 15-18, W= 4.87, **p = 0.002** |
| | | | 15-18 years | 418 | 1.97 | | | | | 11-14 vs. 15-18, W= 3.03, n.s. |
| | | female | 6-10 years | 26 | 1.31 | 11.55 | 2 | **0.003** | 0.02 | 6-10 < 11-14 , W= 4.12, **p = 0.010** |
| | | | 11-14 years | 205 | 1.88 | | | | | 6-10 < 15-18, W= 4.72, **p = 0.002** |
| | | | 15-18 years | 296 | 1.98 | | | | | 11-14 vs. 15-18, W= 1.51, n.s. |
| | | male | 6-10 years | 29 | 1.66 | 5.18 | 2 | 0.075 | 0.02 | 6-10 vs. 11-14 , W= 0.45, n.s. |
| | | | 11-14 years | 117 | 1.70 | | | | | 6-10 vs. 15-18, W= 2.16, n.s. |
| | | | 15-18 years | 122 | 1.96 | | | | | 11-14 vs. 15-18, W= 2.91, n.s. |
| Do you usually sleep during the day? | [1] yes<br>[2] no | all | 6-10 years | 375 | 1.99 | 189.13 | 2 | **< 0.001** | 0.08 | 6-10 > 11-14 , W= -6.91, **p < 0.001** |
| | | | 11-14 years | 952 | 1.92 | | | | | 6-10 > 15-18, W= -15.04, **p < 0.001** |
| | | | 15-18 years | 930 | 1.75 | | | | | 11-14 > 15-18, W= -14.64, **p < 0.001** |
| | | female | 6-10 years | 187 | 1.99 | 116.91 | 2 | **< 0.001** | 0.08 | 6-10 > 11-14 , W= -6.06, **p < 0.001** |
| | | | 11-14 years | 576 | 1.89 | | | | | 6-10 > 15-18, W= -11.68, **p < 0.001** |
| | | | 15-18 years | 676 | 1.70 | | | | | 11-14 > 15-18, W= -11.47, **p < 0.001** |
| | | male | 6-10 years | 188 | 1.98 | 55.43 | 2 | **< 0.001** | 0.07 | 6-10 vs. 11-14 , W= -2.96, n.s. |
| | | | 11-14 years | 376 | 1.94 | | | | | 6-10 > 15-18, W= -8.32, **p < 0.001** |
| | | | 15-18 years | 254 | 1.79 | | | | | 11-14 > 15-18, W= -8.04, **p < 0.001** |

| | response option | | age group | n | M | $\chi^2$ | df | p | ε² | DSCF |
|---|---|---|---|---|---|---|---|---|---|---|
| Do you usually sleep during the day? | [1] I nap from time to time<br>[2] I nap regularly<br>[3] I often fall asleep unintentionally during the day | all | 6-10 years | 6 | 1.25 | 0.96 | 2 | 0.620 | 0.00 | 6-10 vs. 11-14 , W= 1.26, n.s. |
| | | | 11-14 years | 88 | 1.58 | | | | | 6-10 vs. 15-18, W= 1.40, n.s. |
| | | | 15-18 years | 258 | 1.66 | | | | | 11-14 vs. 15-18, W= 0.15, n.s. |
| | | female | 6-10 years | 2 | 1.00 | 1.69 | 2 | 0.429 | 0.01 | 6-10 vs. 11-14 , W= 1.58, n.s. |
| | | | 11-14 years | 65 | 1.72 | | | | | 6-10 vs. 15-18, W= 1.52, n.s. |
| | | | 15-18 years | 204 | 1.61 | | | | | 11-14 vs. 15-18, W= -1.01, n.s. |
| | | male | 6-10 years | 4 | 1.50 | 2.51 | 2 | 0.285 | 0.03 | 6-10 vs. 11-14 , W= -0.06, n.s. |
| | | | 11-14 years | 23 | 1.43 | | | | | 6-10 vs. 15-18, W= 0.86, n.s. |
| | | | 15-18 years | 54 | 1.70 | | | | | 11-14 vs. 15-18, W= 2.15, n.s. |
| Did the frequency of napping change during COVID-19? | [1] yes<br>[2] no | all | 6-10 years | 375 | 1.94 | 192.28 | 2 | **< 0.001** | 0.09 | 6-10 > 11-14 , W= -7.05, **p < 0.001** |
| | | | 11-14 years | 952 | 1.85 | | | | | 6-10 > 15-18, W= -16.09, **p < 0.001** |
| | | | 15-18 years | 930 | 1.66 | | | | | 11-14 > 15-18, W= -14.60, **p < 0.001** |
| | | female | 6-10 years | 187 | 1.90 | 112.30 | 2 | **< 0.001** | 0.08 | 6-10 > 11-14 , W= -4.39, **p = 0.005** |
| | | | 11-14 years | 576 | 1.81 | | | | | 6-10 > 15-18, W= -11.48, **p < 0.001** |
| | | | 15-18 years | 676 | 1.58 | | | | | 11-14 > 15-18, W= -11.88, **p < 0.001** |
| | | male | 6-10 years | 188 | 1.98 | 54.06 | 2 | **< 0.001** | 0.07 | 6-10 > 11-14 , W= -5.23, **p < 0.001** |
| | | | 11-14 years | 376 | 1.89 | | | | | 6-10 > 15-18, W= -9.49, **p < 0.001** |
| | | | 15-18 years | 254 | 1.74 | | | | | 11-14 > 15-18, W= -6.68, **p < 0.001** |
| | [1] I nap a lot more than before COVID-19<br>[2] I nap more than before COVID-19<br>[3] I nap less than before COVID-19<br>[4] I nap a lot less than before COVID-19 | all | 6-10 years | 22 | 2.86 | 2.94 | 2 | 0.230 | 0.01 | 6-10 vs. 11-14 , W= 2.47, n.s. |
| | | | 11-14 years | 154 | 3.15 | | | | | 6-10 vs. 15-18, W= 2.04, n.s. |
| | | | 15-18 years | 346 | 3.08 | | | | | 11-14 vs. 15-18, W= -0.96, n.s. |
| | | female | 6-10 years | 18 | 2.72 | 2.20 | 2 | 0.332 | 0.01 | 6-10 vs. 11-14 , W= 2.16, n.s. |
| | | | 11-14 years | 112 | 3.08 | | | | | 6-10 vs. 15-18, W= 1.99, n.s. |
| | | | 15-18 years | 281 | 3.04 | | | | | 11-14 vs. 15-18, W= -0.24, n.s. |
| | | male | 6-10 years | 4 | 3.00 | 1.08 | 2 | 0.582 | 0.01 | 6-10 vs. 11-14 , W= 0.98, n.s. |
| | | | 11-14 years | 42 | 3.21 | | | | | 6-10 vs. 15-18, W= 0.57, n.s. |
| | | | 15-18 years | 65 | 3.11 | | | | | 11-14 vs. 15-18, W= -1.27, n.s. |

**Notes:** effect size ε²: < 0.04 very small to small effect; 0.04-0.16 medium effect; 0.16-0.64 large effect; > 0.64 very large effect; DSCF: Dwass-Steel-Critchlow-Fligner test corrects for multiple comparisons.

**Table S10.** Comparison between males and females for changes in time spent in bed, regularity of bedtimes and daytime sleep/napping.

| | response option | age group | N | M | sex | n | M | U | p | r |
|---|---|---|---|---|---|---|---|---|---|---|
| How did the time you usually spend in bed change? | [1] a lot shorter<br>[2] considerably shorter<br>[3] sligthly shorter<br>[4] no change<br>[5] slightly longer<br>[6] considerably longer<br>[7] a lot longer | 6-10 years | 375 | 4.19 | female<br>male | 187<br>188 | 4.22<br>4.15 | 16944.0 | 0.510 | 0.04 |
| | | 11-14 years | 952 | 4.43 | female<br>male | 576<br>376 | 4.46<br>4.39 | 103284.0 | 0.215 | 0.05 |
| | | 15-18 years | 930 | 4.72 | female<br>male | 676<br>254 | 4.94<br>4.49 | 71158.0 | **< 0.001** | 0.17 |
| | | 6-18 years | 2257 | 4.51 | female<br>male | 1439<br>818 | 4.66<br>4.36 | 50816.0 | **< 0.001** | 0.14 |
| Do you/did you go to bed at the same time every day (+/- 30min)? This question refers to the time during COVID-19 as well as to the normal state before COVID-19 | [1] yes<br>[2] no | 6-10 years | 375 | 1.15 | female<br>male | 187<br>188 | 1.14<br>1.15 | 17310.5 | 0.678 | 0.02 |
| | | 11-14 years | 952 | 1.34 | female<br>male | 576<br>376 | 1.36<br>1.31 | 103444.0 | 0.154 | 0.04 |
| | | 15-18 years | 930 | 1.46 | female<br>male | 676<br>254 | 1.44<br>1.48 | 82335.0 | 0.264 | 0.04 |
| | | 6-18 years | 2257 | 1.35 | female<br>male | 1439<br>818 | 1.37<br>1.33 | 565425.0 | 0.060 | 0.04 |
| | [1] my bedtime is irregular independently of COVID-19<br>[2] my bedtime was regular before and got irregular during COVID-19 [3] my bedtime was irregular before and got regular during COVID-19 | 6-10 years | 55 | 1.49 | female<br>male | 26<br>29 | 1.31<br>1.66 | 307.0 | 0.122 | 0.19 |
| | | 11-14 years | 322 | 1.79 | female<br>male | 205<br>117 | 1.88<br>1.70 | 10865.0 | 0.110 | 0.09 |
| | | 15-18 years | 418 | 1.97 | female<br>male | 296<br>122 | 1.98<br>1.96 | 17870.0 | 0.851 | 0.01 |
| | | 6-18 years | 795 | 1.86 | female<br>male | 527<br>268 | 1.91<br>1.81 | 67225.0 | 0.207 | 0.05 |
| Do you usually sleep during the day? | [1] yes<br>[2] no | 6-10 years | 375 | 1.99 | female<br>male | 187<br>188 | 1.99<br>1.98 | 17392.0 | 0.416 | 0.01 |
| | | 11-14 years | 952 | 1.92 | female<br>male | 576<br>376 | 1.89<br>1.94 | 102692.0 | **0.007** | 0.05 |
| | | 15-18 years | 930 | 1.75 | female<br>male | 676<br>254 | 1.70<br>1.79 | 78196.0 | **0.007** | 0.09 |
| | | 6-18 years | 2257 | 1.86 | female<br>male | 1439<br>818 | 1.81<br>1.90 | 535992.0 | **< 0.001** | 0.09 |

| | response option | age group | N | M | sex | n | M | U | p | r |
|---|---|---|---|---|---|---|---|---|---|---|
| Do you usually sleep during the day? | [1] I nap from time to time<br>[2] I nap regularly<br>[3] I often fall asleep unintentionally during the day | 6-10 years | 6 | 1.25 | female<br>male | 2<br>4 | 1.00<br>1.50 | 3.0 | 0.724 | 0.25 |
| | | 11-14 years | 88 | 1.58 | female<br>male | 65<br>23 | 1.72<br>1.43 | 633.0 | 0.200 | 0.15 |
| | | 15-18 years | 258 | 1.66 | female<br>male | 204<br>54 | 1.61<br>1.70 | 5092.0 | 0.332 | 0.08 |
| | | 6-18 years | 352 | 1.63 | female<br>male | 271<br>81 | 1.63<br>1.62 | 10938.0 | 0.957 | 0.00 |
| Did the frequency of napping change during COVID-19? | [1] yes<br>[2] no | 6-10 years | 375 | 1.94 | female<br>male | 187<br>188 | 1.90<br>1.98 | 16260.0 | **0.002** | 0.07 |
| | | 11-14 years | 952 | 1.85 | female<br>male | 576<br>376 | 1.81<br>1.89 | 99328.0 | **< 0.001** | 0.08 |
| | | 15-18 years | 930 | 1.66 | female<br>male | 676<br>254 | 1.58<br>1.74 | 72135.0 | **< 0.001** | 0.16 |
| | | 6-18 years | 2257 | 1.79 | female<br>male | 1439<br>818 | 1.71<br>1.86 | 500317.0 | **< 0.001** | 0.15 |
| | [1] I nap a lot more than before COVID-19<br>[2] I nap more than before COVID-19<br>[3] I nap less than before COVID-19<br>[4] I nap a lot less than before COVID-19 | 6-10 years | 22 | 2.86 | female<br>male | 18<br>4 | 2.72<br>3.00 | 31.5 | 0.708 | 0.13 |
| | | 11-14 years | 154 | 3.15 | female<br>male | 112<br>42 | 3.08<br>3.21 | 2068.0 | 0.177 | 0.12 |
| | | 15-18 years | 346 | 3.08 | female<br>male | 281<br>65 | 3.04<br>3.11 | 8783.0 | 0.586 | 0.04 |
| | | 6-18 years | 522 | 3.09 | female<br>male | 411<br>111 | 3.04<br>3.14 | 21151.0 | 0.179 | 0.07 |

**Notes:** effect size r = biserial rank correlation: < 0.28 very small to small effect; 0.28 – 0.43 medium effect; > 0.43 large effect

**Table S11.** Changes in physical activity, daylight exposure, and usage of media devices.

| | | 6-10 years | | | | | 11-14 years | | | | | 15-18 years | | | | |
|---|---|---|---|---|---|---|---|---|---|---|---|---|---|---|---|---|
| | | | | | 95% CI | | | | | 95% CI | | | | | 95% CI | |
| | response option | n | N | % | Lower | Upper | n | N | % | Lower | Upper | n | N | % | Lower | Upper |
| Did the extent of physical activity change during COVID-19 compared to before? | [1] yes | 281 | 375 | 74.9 | 70.23 | 79.24 | 729 | 952 | 76.6 | 73.75 | 79.23 | 737 | 930 | 79.2 | 76.5 | 81.81 |
| | [2] no | 94 | 375 | 25.1 | 20.76 | 29.77 | 223 | 952 | 23.4 | 20.77 | 26.25 | 193 | 930 | 20.8 | 18.19 | 23.50 |
| | [1] a lot less active than before COVID-19 | 75 | 281 | 26.7 | 21.61 | 32.27 | 208 | 729 | 28.5 | 25.28 | 31.96 | 217 | 737 | 29.4 | 26.17 | 32.88 |
| | [2] less active than before COVID-19 | 174 | 281 | 61.9 | 55.97 | 67.62 | 351 | 729 | 48.1 | 44.47 | 51.85 | 278 | 737 | 37.7 | 34.21 | 41.33 |
| | [3] more active than before COVID-19 | 23 | 281 | 8.2 | 5.26 | 12.03 | 107 | 729 | 14.7 | 12.19 | 17.46 | 168 | 737 | 22.8 | 19.81 | 26.00 |
| | [4] a lot more active than before COVID-19 | 9 | 281 | 3.2 | 1.48 | 5.99 | 63 | 729 | 8.6 | 6.70 | 10.92 | 74 | 737 | 10.0 | 7.97 | 12.44 |
| How much time do you spend outdoors (including time spent on balcony and in the garden) in daylight during COVID-19 compared to before? | [1] a lot less than before COVID-19 | 29 | 375 | 7.7 | 5.24 | 10.92 | 132 | 951 | 13.9 | 11.75 | 16.24 | 167 | 930 | 18.0 | 15.54 | 20.58 |
| | [2] less than before COVID-19 | 99 | 375 | 26.4 | 22.01 | 31.17 | 283 | 951 | 29.8 | 26.87 | 32.78 | 290 | 930 | 31.2 | 28.22 | 34.27 |
| | [3] no change | 175 | 375 | 46.7 | 41.53 | 51.86 | 299 | 951 | 31.4 | 28.50 | 34.5 | 250 | 930 | 26.9 | 24.06 | 29.86 |
| | [4] more than before COVID-19 | 60 | 375 | 16.0 | 12.44 | 20.11 | 169 | 951 | 17.8 | 15.39 | 20.35 | 166 | 930 | 17.8 | 15.44 | 20.47 |
| | [5] a lot more than before COVID-19 | 12 | 375 | 3.2 | 1.66 | 5.52 | 68 | 951 | 7.2 | 5.60 | 8.98 | 57 | 930 | 6.1 | 4.68 | 7.87 |
| Do you spend more time with smartphone/TV/gaming console/tablet/PC etc during COVID-19 compared to before? | [1] yes | 279 | 375 | 74.4 | 69.67 | 78.74 | 806 | 952 | 84.7 | 82.22 | 86.9 | 840 | 930 | 90.3 | 88.24 | 92.15 |
| | [2] no | 96 | 375 | 25.6 | 21.26 | 30.33 | 146 | 952 | 15.3 | 13.11 | 17.78 | 90 | 930 | 9.7 | 7.85 | 11.76 |
| | [1] a lot less than before COVID-19 | | | | | | 2 | 806 | 0.2 | 0.03 | 0.89 | 2 | 840 | 0.2 | 0.03 | 0.86 |
| | [2] less than before COVID-19 | | | | | | 10 | 806 | 1.2 | 0.60 | 2.27 | 5 | 840 | 0.6 | 0.19 | 1.38 |
| | [3] more than before COVID-19 | 203 | 279 | 72.8 | 67.13 | 77.9 | 487 | 806 | 60.4 | 56.95 | 63.82 | 442 | 840 | 52.6 | 49.18 | 56.04 |
| | [4] a lot more than before COVID-19 | 76 | 279 | 27.2 | 22.1 | 32.87 | 307 | 806 | 38.1 | 34.72 | 41.54 | 391 | 840 | 46.5 | 43.13 | 49.99 |

**Notes:** CI, 95% confidence interval with lower and upper border; empty spaces: response option was not chosen in this age group

**Table S12**. Comparison between age groups for changes in physical activity, daylight exposure and usage of media devices.

| | response option | | age group | n | M | $\chi^2$ | df | p | ε² | DSCF |
|---|---|---|---|---|---|---|---|---|---|---|
| Did the extent of physical activity change during COVID-19 compared to before? | [1] yes<br>[2] no | all | 6-10 years | 375 | 1.25 | 3.49 | 2 | 0.175 | 0.00 | 6-10 vs. 11-14 , W= -0.89, n.s. |
| | | | 11-14 years | 952 | 1.24 | | | | | 6-10 vs. 15-18, W= -2.41, n.s. |
| | | | 15-18 years | 930 | 1.21 | | | | | 11-14 vs. 15-18, W= -1.97, n.s. |
| | | female | 6-10 years | 187 | 1.27 | 3.95 | 2 | 0.139 | 0.00 | 6-10 vs. 11-14 , W= -1.43, n.s. |
| | | | 11-14 years | 576 | 1.23 | | | | | 6-10 vs. 15-18, W= -2.69, n.s. |
| | | | 15-18 years | 676 | 1.20 | | | | | 11-14 vs. 15-18, W= -1.71, n.s. |
| | | male | 6-10 years | 188 | 1.23 | 0.31 | 2 | 0.858 | 0.00 | 6-10 vs. 11-14 , W= 0.20, n.s. |
| | | | 11-14 years | 376 | 1.24 | | | | | 6-10 vs. 15-18, W= -0.48, n.s. |
| | | | 15-18 years | 254 | 1.22 | | | | | 11-14 vs. 15-18, W= -0.78, n.s. |
| | [1] I am a lot less active than before COVID-19<br>[2] I am less active than before COVID-19<br>[3] I am more active than before COVID-19<br>[4] I am a lot more active than before COVID-19 | all | 6-10 years | 281 | 1.88 | 12.76 | 2 | **0.002** | 0.01 | 6-10 vs. 11-14 , W= 2.81, n.s. |
| | | | 11-14 years | 729 | 2.03 | | | | | 6-10 < 15-18, W= 4.97, **p = 0.001** |
| | | | 15-18 years | 737 | 2.09 | | | | | 11-14 vs. 15-18, W= 2.79, n.s. |
| | | female | 6-10 years | 137 | 1.88 | 10.67 | 2 | **0.005** | 0.01 | 6-10 vs. 11-14 , W= 2.52, n.s. |
| | | | 11-14 years | 443 | 2.08 | | | | | 6-10 < 15-18, W= 4.49, **p = 0.004** |
| | | | 15-18 years | 539 | 2.19 | | | | | 11-14 vs. 15-18, W= 2.58, n.s. |
| | | male | 6-10 years | 144 | 1.88 | 0.65 | 2 | 0.722 | 0.00 | 6-10 vs. 11-14 , W= 1.11, n.s. |
| | | | 11-14 years | 286 | 1.97 | | | | | 6-10 vs. 15-18, W= 0.95, n.s. |
| | | | 15-18 years | 198 | 1.99 | | | | | 11-14 vs. 15-18, W= 0.01, n.s. |
| How much time do you spend otudoors (including time spent on balcony and in the garden) in daylight during COVID-19 compared to before? | [1] a lot less than before COVID-19<br>[2] less than before COVID-19<br>[3] no change<br>[4] more than before COVID-19<br>[5] a lot more than before COVID-19 | all | 6-10 years | 375 | 2.81 | 11.37 | 2 | **0.003** | 0.01 | 6-10 vs. 11-14 , W= -1.96, n.s. |
| | | | 11-14 years | 952 | 2.75 | | | | | 6-10 > 15-18, W= -4.52, **p= 0.004** |
| | | | 15-18 years | 930 | 2.59 | | | | | 11-14 > 15-18, W= -3.22, p= 0.059 |
| | | female | 6-10 years | 187 | 2.86 | 5.90 | 2 | 0.052 | 0.00 | 6-10 vs. 11-14 , W= -2.26, n.s. |
| | | | 11-14 years | 576 | 2.74 | | | | | 6-10 > 15-18, W= -3.49, **p= 0.037** |
| | | | 15-18 years | 676 | 2.68 | | | | | 11-14 vs. 15-18, W= -1.46, n.s. |
| | | male | 6-10 years | 188 | 2.76 | 9.93 | 2 | **0.007** | 0.01 | 6-10 vs. 11-14 , W= -0.47, n.s. |
| | | | 11-14 years | 376 | 2.75 | | | | | 6-10 > 15-18, W= -3.80, **p= 0.020** |
| | | | 15-18 years | 254 | 2.50 | | | | | 11-14 vs. 15-18, W= -3.92, **p= 0.016** |
| Do you spend more time with smarthpone/TV/gaming console/tablet/PC etc. during COVID-19 compared to before? | [1] yes<br>[2] no | all | 6-10 years | 375 | 1.26 | 54.50 | 2 | **< 0.001** | 0.02 | 6-10 > 11-14 , W= -6.16, **p < 0.001** |
| | | | 11-14 years | 952 | 1.15 | | | | | 6-10 > 15-18, W= -10.53, **p < 0.001** |
| | | | 15-18 years | 930 | 1.11 | | | | | 11-14 > 15-18, W= -5.24, **p < 0.001** |
| | | female | 6-10 years | 187 | 1.29 | 55.37 | 2 | **< 0.001** | 0.04 | 6-10 > 11-14 , W= -5.99, **p < 0.001** |
| | | | 11-14 years | 576 | 1.15 | | | | | 6-10 > 15-18, W= -10.68, **p < 0.001** |
| | | | 15-18 years | 676 | 1.08 | | | | | 11-14 > 15-18, W= -5.45, **p < 0.001** |
| | | male | 6-10 years | 188 | 1.22 | 6.61 | 2 | **0.037** | 0.01 | 6-10 vs. 11-14 , W= -2.78, n.s. |
| | | | 11-14 years | 376 | 1.15 | | | | | 6-10 > 15-18, W= -3.47, **p = 0.038** |
| | | | 15-18 years | 254 | 1.13 | | | | | 11-14 vs. 15-18, W= -1.08, n.s. |

| | response option | | age group | n | M | $\chi^2$ | df | p | ε² | DSCF |
|---|---|---|---|---|---|---|---|---|---|---|
| Do you spend more time with smarthpone/TV/gaming console/tablet/PC etc. during COVID-19 compared to before? | [1] a lot less than before COVID-19 [2] less than before COVID-19 [3] more than before COVID-19 [4] a lot more than before COVID-19 | all | 6-10 years | 279 | 3.28 | 33.02 | 2 | **< 0.001** | 0.02 | 6-10 < 11-14 , W= 4.12, **p = 0.010** |
| | | | 11-14 years | 806 | 3.37 | | | | | 6-10 < 15-18, W= 7.72, **p < 0.001** |
| | | | 15-18 years | 840 | 3.47 | | | | | 11-14 < 15-18, W= 5.03, **p = 0.001** |
| | | female | 6-10 years | 132 | 3.27 | 18.11 | 2 | **< 0.001** | 0.01 | 6-10 vs. 11-14 , W= 3.05, n.s. |
| | | | 11-14 years | 487 | 3.36 | | | | | 6-10 < 15-18, W= 5.53, **p < 0.001** |
| | | | 15-18 years | 619 | 3.45 | | | | | 11-14 < 15-18, W= 3.81, **p = 0.020** |
| | | male | 6-10 years | 147 | 3.28 | 15.43 | 2 | **< 0.001** | 0.02 | 6-10 vs. 11-14 , W= 2.79, n.s. |
| | | | 11-14 years | 319 | 3.37 | | | | | 6-10 < 15-18, W= 5.47, **p < 0.001** |
| | | | 15-18 years | 221 | 3.48 | | | | | 11-14 < 15-18, W= 3.47, **p = 0.038** |

**Notes:** effect size ε²: < 0.04 very small to small effect; 0.04-0.16 medium effect; 0.16-0.64 large effect; > 0.64 very large effect; DSCF: Dwass-Steel-Critchlow-Fligner test corrects for multiple comparisons.

**Table S13.** Comparison between males and females for changes in physical activity, daylight exposure and usage of media devices.

| | response option | age group | N | M | sex | n | M | U | p | r |
|---|---|---|---|---|---|---|---|---|---|---|
| Did the extent of physical activity change during COVID-19 compared to before? | [1] yes [2] no | 6-10 years | 375 | 1.25 | female | 187 | 1.27 | 16992.0 | 0.457 | 0.03 |
| | | | | | male | 188 | 1.23 | | | |
| | | 11-14 years | 952 | 1.24 | female | 576 | 1.23 | 107372.0 | 0.763 | 0.01 |
| | | | | | male | 376 | 1.24 | | | |
| | | 15-18 years | 930 | 1.21 | female | 676 | 1.20 | 84323.0 | 0.551 | 0.02 |
| | | | | | male | 254 | 1.22 | | | |
| | | 6-18 years | 2257 | 1.23 | female | 1439 | 1.22 | 582726.0 | 0.589 | 0.01 |
| | | | | | male | 818 | 1.23 | | | |
| | [1] I am a lot less active than before COVID-19 [2] I am less active than before COVID-19 [3] I am more active than before COVID-19 [4] I am a lot more active than before COVID-19 | 6-10 years | 281 | 1.88 | female | 137 | 1.88 | 9786.5 | 0.896 | 0.01 |
| | | | | | male | 144 | 1.88 | | | |
| | | 11-14 years | 729 | 2.03 | female | 443 | 2.08 | 59717.0 | 0.159 | 0.06 |
| | | | | | male | 286 | 1.97 | | | |
| | | 15-18 years | 737 | 2.09 | female | 539 | 2.19 | 47288.0 | **0.013** | 0.11 |
| | | | | | male | 198 | 1.99 | | | |
| | | 6-18 years | 1747 | 2.03 | female | 1119 | 2.11 | 320832.0 | **0.001** | 0.09 |
| | | | | | male | 628 | 1.95 | | | |
| How much time do you spend otudoors (including time spent on balcony and in the garden) in daylight during COVID-19 compared to before? | [1] a lot less than before COVID-19 [2] less than before COVID-19 [3] no change [4] more than before COVID-19 [5] a lot more than before COVID-19 | 6-10 years | 375 | 2.81 | female | 187 | 2.86 | 16338.0 | 0.207 | 0.07 |
| | | | | | male | 188 | 2.76 | | | |
| | | 11-14 years | 952 | 2.75 | female | 576 | 2.74 | 107457.0 | 0.892 | 0.01 |
| | | | | | male | 376 | 2.75 | | | |
| | | 15-18 years | 930 | 2.59 | female | 676 | 2.68 | 78808.0 | **0.046** | 0.08 |
| | | | | | male | 254 | 2.50 | | | |
| | | 6-18 years | 2257 | 2.70 | female | 1439 | 2.73 | 573831.0 | 0.329 | 0.02 |
| | | | | | male | 818 | 2.67 | | | |

| | response option | age group | N | M | sex | n | M | U | p | r |
|---|---|---|---|---|---|---|---|---|---|---|
| Do you spend more time with smarthpone/TV/gaming console/tablet/PC etc. during COVID-19 compared to before? | [1] yes<br>[2] no | 6-10 years | 375 | 1.26 | female<br>male | 187<br>188 | 1.29<br>1.22 | 16241.5 | 0.092 | 0.08 |
| | | 11-14 years | 952 | 1.15 | female<br>male | 576<br>376 | 1.15<br>1.15 | 107972.0 | 0.903 | 0.00 |
| | | 15-18 years | 930 | 1.11 | female<br>male | 676<br>254 | 1.08<br>1.13 | 81937.0 | **0.036** | 0.05 |
| | | 6-18 years | 2257 | 1.15 | female<br>male | 1439<br>818 | 1.14<br>1.16 | 576506.0 | 0.187 | 0.02 |
| | [1] a lot less than before COVID-19<br>[2] less than before COVID-19<br>[3] more than before COVID-19<br>[4] a lot more than before COVID-19 | 6-10 years | 279 | 3.28 | female<br>male | 132<br>147 | 3.27<br>3.28 | 9568.5 | 0.798 | 0.01 |
| | | 11-14 years | 806 | 3.37 | female<br>male | 487<br>319 | 3.36<br>3.37 | 77295.0 | 0.890 | 0.00 |
| | | 15-18 years | 840 | 3.47 | female<br>male | 619<br>221 | 3.45<br>3.48 | 66300.0 | 0.435 | 0.03 |
| | | 6-18 years | 1925 | 3.39 | female<br>male | 1238<br>687 | 3.39<br>3.38 | 420714.0 | 0.650 | 0.01 |

**Notes:** effect size r = biserial rank correlation: < 0.28 very small to small effect; 0.28 – 0.43 medium effect; > 0.43 large effect
